# The sustained increase of cardiovascular risk following COPD exacerbations: meta-analyses of the EXACOS-CV studies

**DOI:** 10.1183/23120541.01091-2024

**Published:** 2025-06-16

**Authors:** Clementine Nordon, Sami O. Simons, Jonathan Marshall, Hana Müllerová, Michael Pollack, Camilla Bengtsson, Fabian Hoti, Muriel Lobier, Aaro Salosensaari, Ana Cristina Santos, Claus Franz Vogelmeier, Kirsty Rhodes

**Affiliations:** 1 Biopharmaceuticals Medical, AstraZeneca, Cambridge, UK; 2 Department of Respiratory Medicine, NUTRIM Institute of Nutrition and Translational Research in Metabolism, Maastricht University Medical Centre+, Maastricht, the Netherlands; 3 Biopharmaceuticals Medical, AstraZeneca, Wilmington, DE, USA; 4 IQVIA, Solna, Sweden; 5 IQVIA, Espoo, Finland; 6 IQVIA, Turku, Finland; 7 IQVIA, Oeiras, Portugal; 8 Pulmonary and Critical Care Medicine, German Center for Lung Research, Marburg, Germany

## Abstract

**Background:**

The EXAcerbations of COPD and their OutcomeS on CardioVascular disease (EXACOS-CV) multi-database studies have consistently shown an increased risk of serious cardiovascular event following COPD exacerbations, but with some risk temporality variations. EXACOS-CV results were meta-analysed to increase their generalisability and improve precision.

**Methods:**

Studies conducted in Canada, the United States, Germany, the Netherlands, Spain, Italy, Japan and England were meta-analysed, amounting to 1 030 875 individuals. Generally, each study included individuals aged ≥40 years with a COPD diagnosis in 2014–2019; primary outcome was the composite of non-fatal acute coronary syndrome, decompensated heart failure, ischaemic stroke, arrhythmias and all-cause death. Pooled hazard ratios (HR_p_) of risks in post-exacerbation periods (*versus* periods outside exacerbations) were obtained through random effects meta-analysis.

**Results:**

Time periods following an exacerbation (any severity) were associated with increased and sustained risks of the composite outcome: HR_p_ 10.22 (95% CI 5.34–19.57) in days 1–7 and HR_p_ 1.24 (95% CI 1.09–1.40) in days 181–365. Risks were elevated for 6 months (HR_p_ 1.25, 95% CI 1.01–1.55 in days 31–180) and 1 year (HR_p_ 1.48, 95% CI 1.11–1.96 in days 181–365) following a moderate or a severe exacerbation, respectively. In newly diagnosed individuals, risks were increased until days 31–180: HR_p_ 1.66 (95% CI 1.14–2.42) and HR_p_ 1.61 (95% CI 1.28–2.02) following the first and the second post-diagnosis exacerbation, respectively.

**Conclusion:**

Risk of severe cardiovascular events is sustainably increased following an exacerbation of COPD, even early and moderate ones. Cardiopulmonary risk reduction should be a global core target of COPD management.

## Shareable abstract

**Across countries with diverse health systems and populations, cardiovascular event or death risk increased 10-fold in the week following COPD exacerbation onset and was sustained for 1 year. COPD management should target cardiopulmonary risk reduction.** https://bit.ly/3UXGiY2

## Introduction

COPD is characterised by chronic respiratory symptoms due to abnormalities of the airways and/or alveoli that cause persistent, often progressive airflow obstruction [1]. Exacerbations of COPD negatively impact prognosis, leading to more frequent/severe exacerbations, accelerated loss of lung function and increased risk of death [2]. People living with COPD have a two- to three-fold increased risk of cardiovascular disease [3] including a two to five times higher risk of ischaemic heart disease, cardiac dysrhythmia, heart failure, diseases of the pulmonary circulation and diseases of the arteries [4].

Furthermore, COPD is associated with an increased risk for serious cardiopulmonary events including respiratory events, such as exacerbations, and severe cardiovascular events [3]. This excess cardiopulmonary risk can be understood as the chance of an individual with COPD having serious respiratory and/or cardiovascular outcomes [5]. Recently, risk ratios of acute myocardial infarction and stroke in the 3 months following an exacerbation of COPD (compared with none) were estimated at 2.43 (95% confidence interval (CI) 1.40–4.20) and 1.68 (95% CI 1.19–2.38) [6].

The EXAcerbations of COPD and their OutcomeS on CardioVascular disease (EXACOS-CV) was a multi-database observational study programme exploring the risk of severe cardiovascular events by characterising the temporal dynamics of this increased risk for a wide range of outcomes and time periods [7–12]. The association between periods of time following an exacerbation of COPD and the risk of a severe cardiovascular event was explored, for the first time, in newly diagnosed COPD [8–14]. Across all individual studies, an association was found between post-exacerbation time periods (*versus* no exacerbation) and an increased risk of a severe cardiovascular (CV) event. However, there were variations in risk increase temporality among individual studies.

Meta-analysis provides an opportunity to synthetise an individual estimate across studies for the same research question. The aim of this study was to pool evidence generated by the EXACOS-CV studies, quantifying the association between the risk of severe cardiovascular events (non-fatal severe cardiovascular events or all-cause death) and time periods following: 1) an exacerbation of COPD of any severity; 2) a moderate and a severe exacerbation of COPD separately; and 3) a first and a second exacerbation of COPD in people newly diagnosed with COPD.

## Methods

We included in this meta-analysis the EXACOS-CV studies conducted in Germany [12], Canada [9], the Netherlands [11], Spain [13], Italy [14], Japan, the UK [10] and the USA [8] using a common protocol as the basis of the design and analysis for the individual studies [7]. We have previously performed a systematic literature review [6], and the methods are described in supplementary material 1.

### EXACOS-CV study designs

Generally, each local study included individuals aged ≥40 years with a COPD diagnosis during the inclusion period from 1 January 2014 to 31 December 2018/2019 (specific inclusion periods given in supplementary table S1). In the common protocol, cohort entry and start of follow-up was the first identified COPD diagnosis during the study period. In the USA and the UK, index date and start of follow-up was the first exacerbation of COPD. A minimum of 1 year of data availability before cohort entry or index date in the data sources was required. Individuals were defined as newly diagnosed with COPD in the absence of any COPD code prior to cohort entry or index date. From the cohort entry or index date, each individual was followed until the earliest of: 1) the first outcome of interest; or 2) the end of data availability; or 3) censoring on 31 December 2018/2019.

**FIGURE 1:**
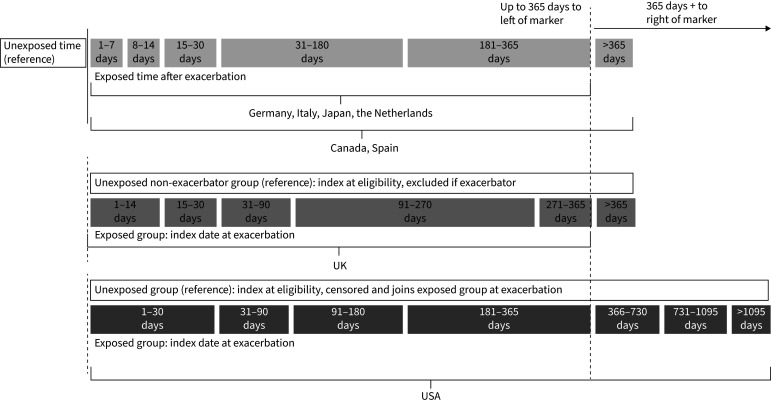
All exposure time periods in the source studies included in the EXACOS-CV meta-analysis. Note: Diagram provided for descriptive purposes only; time blocks are not to scale.

Exacerbations of COPD were exposure events. A moderate exacerbation of COPD was defined as a COPD-related visit to a physician with a dispensation of systemic corticosteroids within the 5 days following the visit and for a duration of <15 days. A severe exacerbation of COPD was defined as hospitalisation of at least one night or a visit to the emergency department with a primary or secondary discharge code for acute exacerbation of COPD. Studies compared exposure periods following a moderate or severe exacerbation of COPD to unexposed time. Exposure periods in each included study are outlined in figure 1 and supplementary table S1. In Germany [12], the Netherlands [11] and Italy [14], unexposed time (the reference period) comprised all follow-up time that was not within 1–365 days following an exacerbation. In Canada [9], Spain [13], Japan and the UK [10], the reference period included only time before a first exacerbation following cohort entry. In the US study [8], unexposed time comprised follow-up time of comparator individuals without a prior exacerbation; comparators were censored upon an exacerbation occurring following cohort entry.

Outcomes were non-fatal severe cardiovascular events, defined as events that required a hospital admission, or all-cause death. Non-fatal severe cardiovascular events were: 1) acute coronary syndrome (ACS) including acute myocardial infarction and unstable angina; 2) decompensated heart failure; 3) ischaemic stroke; and 4) arrhythmias including atrial fibrillation/flutter, resuscitated cardiac arrest and other arrhythmias. The outcome was the first cardiovascular event occurring after cohort entry, considered as the composite of non-fatal cardiovascular event or all-cause death, or for each type of event individually. Endpoints were the time to first occurrence of the outcome.

### Quality assessment

Risk of bias in each individual study was assessed according to the seven bias domains within the Risk Of Bias In Non-randomised Studies – of Exposure (ROBINS-E) tool [15]. Sources of concern are summarised in supplementary table S2.

### Statistical analysis

Each individual study reported confounder-adjusted hazard ratios (HRs) for the association between post-exacerbation time periods and outcomes of interest. Adjusted HRs were directly extracted from published articles or individual study reports.

**FIGURE 2:**
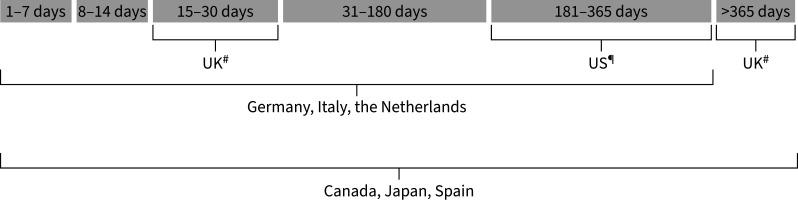
Exposure time periods for composite severe cardiovascular events and individual events after a moderate or a severe exacerbation in the EXACOS-CV studies pooled in the meta-analysis. ACS: acute coronary syndrome; CV: cardiovascular. Note: Diagram provided for descriptive purposes only; time blocks are not to scale. ^#^: exposure time period in the analysis of individual CV events (ACS, decompensated heart failure, ischaemic stroke, arrythmia events); ^¶^: exposure time period in the analysis of composite severe CV events.

We conducted a meta-analysis of pooled exposures and outcomes because we had no access to patient-level data. Individual country results were pooled for the exposure event (*i.e.*, moderate or severe, moderate, severe, first and second exacerbation following a new diagnosis of COPD), exposure time period (figure 2) and outcome when available. Associations were estimated between time periods following: 1) an exacerbation (any severity) and the composite outcome; or 2) each individual type of cardiovascular event; 3) a moderate or a severe exacerbation and the composite outcome; and in people newly diagnosed with COPD 4) a first and second exacerbation and the composite outcome.

Random effects meta-analysis was used to obtain pooled estimates of adjusted HR (HR_p_), based on the inverse variance method. Log-transformed adjusted HRs and their standard errors were used to conduct the meta-analysis. The corresponding standard errors were derived from the reported 95% CIs. The DerSimonian–Laird estimator [16] was used to quantify between-study variance τ^2^, and the method of Jackson was used to compute the 95% CI for τ^2^ [17]. Higgins’ and Thompson's I^2^ statistic was computed to quantify the proportion of variation among estimated HRs that is due to between-study heterogeneity [18]. Note that I^2^ tends towards 100% when number of patients in source studies increases [19]. Analyses were conducted in R (version 4.1.1) with package meta (version 6.5–0) [20].

Forest plots were created to visually inspect for statistical heterogeneity among study results. All individual country-specific results for the same exposure time period and outcome were reported together on the same forest plot and meta-analysed.

## Results

The total sample size across studies included in this meta-analysis was 1 030 875 people living with COPD (table 1). Mean±sd age varied from 63.0±12.0 years old in the USA to 74.0±12.0 in Italy. The proportion of males varied from 44.6% in the USA to 78.2% in Spain. Hypertension was the most common cardiovascular comorbidity at baseline in all studies, with the lowest prevalence reported in the Netherlands (37.0%) and the highest in Germany (75.2%).

**TABLE 1:** Descriptive characteristics at baseline

	Germany	Canada	The Netherlands^#^	Spain	Italy	Japan	UK	USA^#^
**Number in cohort**								
** **Total	126 795	142 787	8 020	24 393	216 864	152 712	213 466	145 838
** **US 1 – exacerbation cohort								145 838
** **US 0 – exacerbation comparator cohort								249 703
**Age years, mean±sd**	66.5±12.0	68.1±12.3	65±11	67.9±11.6	74±12	73.7±11.2	68.8±11.2	63±12
**Male, n (%)**	76 074 (60.0)	73 777 (51.7)	4 223 (53)	19 085 (78.2)	120 257 (55.5)	95 280 (62.4)		65 025 (44.6)
**Female, n (%)**							102 025 (47.8)	
**Cardiovascular risk factors, n (%)**								
** **Obesity	44 285 (34.9)	NA	919 (11)	4 131 (16.9)	6 561 (3.0)	886 (0.6)	58 909 (27.6)	NA
** **Diabetes mellitus Type 2	39 238 (30.9)	27 527 (19.3)	1 076 (13)	4 190 (17.2)	50 548 (23.3)	31 910 (20.9)	38 453 (18.0)	34 066 (23.4)
** **Disorders of lipoprotein metabolism and other dyslipidaemia	71 809 (56.6)	31 346 (22.0)	1 503 (19)	12 263 (50.3)	74 586 (34.4)	54 399 (35.6)	NA	97 733 (67.0)
** **Hypertension	95 378 (75.2)	66 462 (46.5)	3 001 (37)	14 576 (59.8)	164 441 (75.8)	82 945 (54.3)	102 659 (48.1)	99 481 (68.2)
**Comorbidities, n (%)**								
** **Ischaemic heart diseases	46 341 (36.5)	36 386 (25.5)	1 393 (17)	4 345 (17.8)	19 713 (9.1)	39 707 (26.0)	15 089 (7.1)	NA
** **Heart failure	35 371 (27.9)	18 649 (13.1)	547 (7)	4 648 (19.1)	22 618 (10.4)	40 019 (26.2)	3 672 (1.7)^¶^	16 374 (11.2)
** **Pulmonary oedema	1 141 (0.9)	4 152 (2.9)	40 (<0.5)	840 (3.4)	736 (0.3)	547 (0.4)	NA	4 857 (3.3)
** **Pulmonary hypertension	6 085 (4.8)	1 654 (1.2)	32 (<0.5)	833 (3.4)	2 379 (1.1)	988 (0.6)	942 (0.4)^¶^	NA
** **Venous thromboembolism	13 191 (10.4)	3 505 (2.5)	310 (4)	1 833 (7.5)	1 462 (0.7)	5 791 (3.8)	2 889 (1.4)	NA
** **Cerebrovascular disease	29 808 (23.5)	13 276 (9.3)	763 (10)	2 408 (9.9)	16 804 (7.7)	32 876 (21.5)	9 795 (4.6)^¶^	NA
** **Arrhythmia	35 607 (28.1)	22 126 (15.5)	864 (11)	2 765 (11.3)	30 602 (14.1)	27 769 (18.2)	18 036 (8.5)^¶^	14 249 (9.8)
** **Asthma	45 972 (36.3)	6 093 (4.3)	940 (12)	2 490 (10.2)	NA	35 867 (23.5)	27 476 (12.9)	38 720 (26.6)
** **Chronic kidney disease	26 328 (20.8)	15 184 (10.6)	387 (5)	2 598 (10.7)	15 188 (7.0)	13 678 (9.0)	27 874 (13.1)	14 997 (10.3)
** **Severe mental illness	20 218 (15.9)	8 908 (6.2)	1 564 (20)	3 633 (14.9)	31 836 (14.7)	6 150 (4.0)	NA	
** **Anxiety disorder	58 085 (45.8)	23 163 (16.2)	NA	9 248 (37.9)	893 (0.4)	19 810 (13.0)	54 804 (25.7)	58 181 (39.9)
**Medication use in the past 12 months, n (%)**								
** **Cardiac medication (any)	94 112 (74.2)	87 153 (61.0)	3 778 (47)	22 285 (91.4)	NA	26 496 (17.4)	NA	NA
** **Antithrombotic agents	45 989 (36.3)	NA		3704 (15.2)	188 568 (87.0)	79 115 (51.8)		
** **Antiarrhythmics, class I/II	2773 (2.2)	NA		2588 (10.6)			59 325 (27.8)	
** **Digitalis glycosides	4893 (3.9)	NA		NA^+^			1783 (0.8)	
** **Antihypertensives (others)	6611 (5.2)	NA		1311 (5.4)			3090 (1.5)	
** **Diuretics	44 512 (35.1)	NA		10 783 (44.2)				
** **β-blocking agents	52 225 (41.2)	NA		11 712 (48)				
** **Calcium channel blockers	31 841 (25.1)	NA		2774 (11.4)			35 117 (16.5)	
** **Agents acting on the renin–angiotensin system	72 243 (57.0)	NA		12 826 (52.6)				
Metabolic medication (any)	52 583 (41.5)	62 193 (43.6)	2 722 (34)	15 188 (62.3)	NA	NA	NA	NA
** **Lipid-modifying agents	42 175 (33.3)	NA^§^		12 995 (53.3)		45 772 (30.0)		
** **Antidiabetic agents	24 494 (19.3)	NA^§^		4631 (19)			98 264 (46.0)	70 482 (48.3)

NA: not available. ^#^: people newly diagnosed with COPD (results rounded with no decimals); ^¶^: measured in the prior year; ^+^: all antiarrhythmic drugs were counted together; ^§^: all metabolic medications were counted together.

### Risk of a cardiovascular event or death following an exacerbation of COPD

**FIGURE 3:**
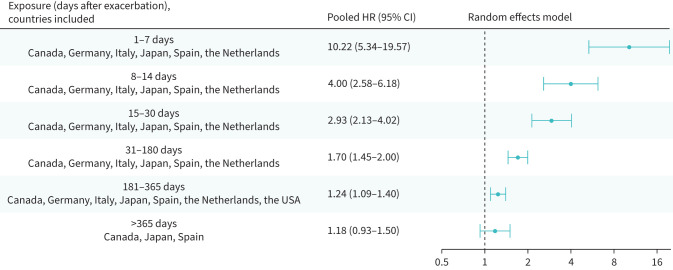
Risk of severe cardiovascular event or all-cause death following an exacerbation of any severity; random effects meta-analysis of adjusted hazard ratios. CI: confidence interval; HR: hazard ratio.

In the 1–7 days following the onset of an exacerbation of any severity, there was a 10-fold increased risk of a severe cardiovascular event or death (HR_p_ 10.22, 95% CI 5.34–19.57) (figure 3). The increased risk was sustained for 1 year, with an incremental decrease of the strength of association, from HR_p_ 4.00 (95% CI 2.58–6.18) in days 8–14 to HR_p_ 1.24 (95% CI 1.09–1.40) in days 181–365. Beyond the year following the onset of an exacerbation, there was no significant risk increase (HR_p_ 1.18, 95% CI 0.93–1.50).

### Risk of individual cardiovascular events and death following an exacerbation

The risks of a hospitalisation for ACS and arrhythmias were increased for up to 6 months following the onset of an exacerbation of any severity (supplementary figures S1–S2). The risks of hospitalisation for decompensated heart failure and ischaemic stroke were increased for up to a year following the onset of an exacerbation (supplementary figures S3–S4). The risk of all-cause death was also increased for up to 1 year following an exacerbation of any severity (supplementary figure S5).

### Risk of a cardiovascular event or death following a moderate or a severe exacerbation

**FIGURE 4:**
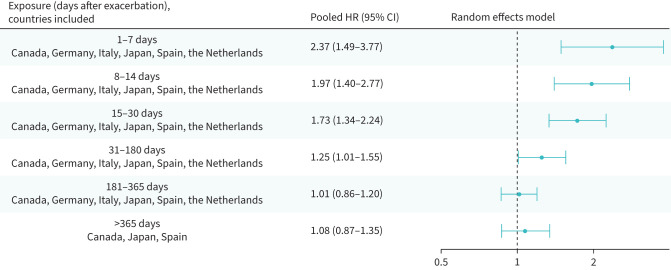
Risk of severe cardiovascular event or all-cause death following a moderate exacerbation; random effects meta-analysis of adjusted hazard ratios (results for the UK and USA not included). CI: confidence interval; HR: hazard ratio.

**FIGURE 5:**
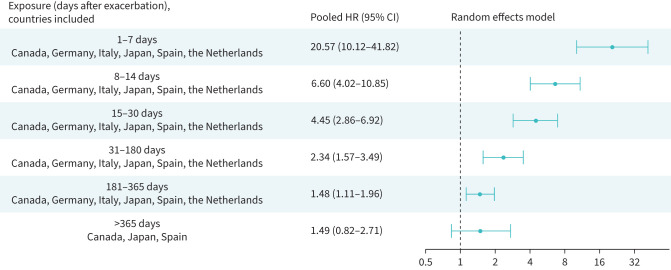
Risk of severe cardiovascular event or all-cause death following a severe exacerbation; random effects meta-analysis of adjusted hazard ratios. CI: confidence interval; HR: hazard ratio.

Following the onset of a moderate exacerbation, all time periods up to 6 months were associated with an increased risk of severe cardiovascular event or death (figure 4). The risk was more than doubled in days 1–7 (HR_p_ 2.37, 95% CI 1.49–3.77), and increased by 25% in days 31–180 (HR_p_ 1.25, 95% CI 1.01–1.55). Following the onset of a severe exacerbation, the risk increase was more pronounced (days 1–7 HR 20.57, CI 10.12–41.82) and sustained for 1 year post-exacerbation (figure 5).

### Risk of a cardiovascular event or death following a first or a second exacerbation in people newly diagnosed with COPD

**FIGURE 6:**
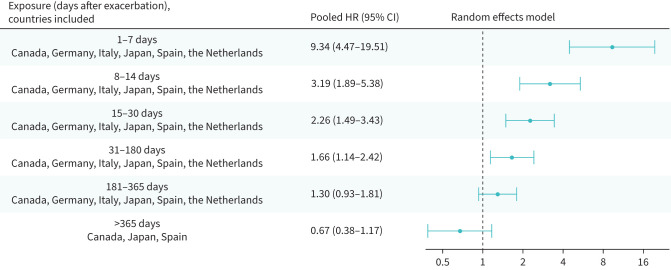
Risk of severe cardiovascular event or all-cause death following a first exacerbation in people newly diagnosed with COPD; random effects meta-analysis of adjusted hazard ratios. CI: confidence interval; HR: hazard ratio.

**FIGURE 7:**
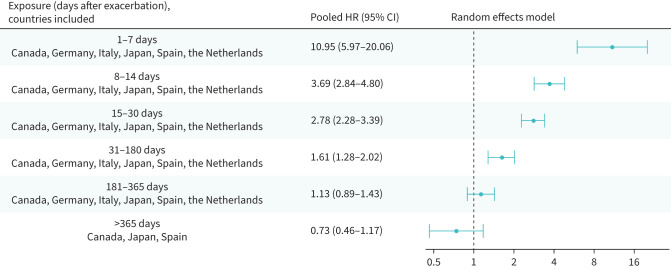
Risk of severe cardiovascular event or all-cause death following a second exacerbation in people newly diagnosed with COPD; random effects meta-analysis of adjusted hazard ratios. CI: confidence interval; HR: hazard ratio.

In individuals with a new diagnosis of COPD, risks of a severe cardiovascular event or death were increased until days 31–180: HR_p_ 1.66 (95% CI 1.14–2.42) and HR_p_ 1.61 (95% CI 1.28–2.02) following the first (figure 6) and the second post-diagnosis exacerbation (figure 7), respectively.

### Statistical heterogeneity among study results

Individual study results consistently showed an immediate increased risk of a severe cardiovascular event or death following an exacerbation that gradually decreased over time. Heterogeneity between studies in the strength of the association between post-exacerbation periods and the risk of a severe cardiovascular event or death was highest in the earlier exposure periods, up to the 30 days exposure period (supplementary figures S6–S15). Between-study variance estimates for the 15–30 days period were highest for the outcome of heart failure decompensation (τ^2^ 0.08, 95% CI 0.04–0.60) and of all-cause death (τ^2^ 0.08, 95% CI 0.05–1.14). Despite the high heterogeneity observed in earlier exposure periods, the direction of the association between post-exacerbation periods and the risk of a severe cardiovascular event or death and the decreasing pattern over time were consistent.

## Discussion

In this meta-analysis of observational cohort studies including more than one million people living with COPD in eight countries, we found an increased risk of a severe cardiovascular event or death which was seen across the spectrum of all cardiovascular events.

### Key findings

An important result of this meta-analysis and the individual studies was that the duration of cardiovascular events risks increased and persisted beyond the immediate period of the exacerbation event. Risks were elevated for 6 months following a moderate exacerbation and for 1 year following a severe exacerbation. In newly diagnosed COPD, risks were elevated for 6 months following either the first or the second post-diagnosis exacerbation, with similar strength of associations. The direction and strength of these associations were present in all studies, irrespective of geographical location or healthcare system, suggesting common biological pathways drive the increased cardiovascular risk of COPD during and after an exacerbation. These findings underscore the syndemics at play between the lungs and heart and the impact of exacerbations on acute decompensation of cardiac disease. Secondly the risk increase was observed for cardiovascular events of a variety of pathophysiological mechanisms, *i.e.*, atherosclerotic events (ischaemic stroke, ACS), arrhythmias and decompensated heart failure. This highlights the multiplicity of effects that COPD and exacerbations exert on the cardiovascular system [21, 22] with mechanisms that include systemic inflammation, hypoxaemia and hypercoagulable state.

### Clinical implications

The immediate period following an exacerbation provides a critical opportunity for cardiac monitoring and COPD treatment adaptation where current implementation of guideline recommended care is lacking [21, 22]. A range of approaches have been suggested including optimisation of COPD maintenance inhaled therapies, relevant vaccinations and addressing common risk factors such as smoking. Additionally, improvement of cardiac disease management should be explored after an exacerbation. However, there remains inconsistency in the guidelines on how to manage comorbid cardiovascular/COPD patients following acute (hospitalised) exacerbations which may contribute to these observed risks.

Our results emphasise the need to focus on prevention of COPD exacerbations by optimising management for patients at the initial COPD diagnosis. Exacerbations may go unnoticed by patients or miscoded as lower-respiratory infections by providers; therefore, it is important that all signs and symptoms be considered when minimising risk.

In the current study, increased cardiovascular risk was found in people newly diagnosed with COPD and following a moderate exacerbation. Considering that most exacerbations are of moderate severity, prevention of exacerbations should be a key therapeutic target from the early course of disease. Early management of COPD with guideline-based care provided by a pulmonologist was recently found to decrease healthcare utilisation for respiratory illness compared to routine general practice care [23]. However, beyond the prevention of exacerbations of COPD, patients with COPD should benefit from integrated care, *i.e.*, be managed holistically [24, 25]. Guidelines and Quality Improvement Initiatives have recently been published emphasising COPD as a risk factor for cardiopulmonary events [26, 27]. There should be a “zero-tolerance” approach to exacerbations by healthcare providers, patients and policy makers and COPD exacerbations should not be seen as an inevitable consequence of living with COPD [28].

### Strengths and limitations

To our knowledge, this is the first meta-analysis of multi-country real-world studies based on a common protocol, which reports on the risk of severe cardiovascular events following both a moderate and severe exacerbation. Previous studies showed an increased risk of severe CV events during the days and months following an exacerbation [6, 29–32], which was confirmed in EXACOS-CV studies. However, they used data from clinical trials (usually selecting more severe patients), or self-controlled designs in which only case patients are included [32, 33]. The results from the EXACOS-CV studies and their meta-analyses expand previous findings by establishing the risk increase also in newly diagnosed patients, in a wide range of CV events, and in relation to both moderate and severe exacerbations. Our results confirm and expand upon previous studies by demonstrating the generalisability of findings to various healthcare systems in several continents and in various high-income countries exposed to different individual and environmental risk factors and at different stages of COPD severities. HRs from all included individual studies were robustly estimated and adjusted for key confounders including prior cardiovascular risk. Following the conduct of the present meta-analysis, a study performed in China [34] – an upper-middle income country – showed an elevated risk of mortality and severe cardiovascular events following severe COPD exacerbation(s). Altogether, these results highlight the significant burden of severe cardiovascular events after an exacerbation of COPD across a diverse range of countries and environments. The inclusion of large population-based cohorts and the use of claims or electronic medical records databases minimised the risk of selection bias.

Limitations should also be addressed. Misclassification of cardiovascular events as primarily respiratory events are possible in the first weeks following an exacerbation owing to similar clinical presentations. This may have contributed to the large effect size observed, *e.g.*, in the first 7 days and for heart failure decompensation. Results concerning the 1–7 days should therefore be interpreted with caution in terms of effect size. Beyond the year following onset of an exacerbation (>365 days time window), data points were available for only three countries (Canada, Spain and the USA) and showed no significant risk increase. However, in the other countries there was no follow-up after 1 year.

Furthermore, unmeasured confounding could not be totally ruled out. For instance, smoking status could not be accounted for in all individual study models due to missing data. We conducted a separate study to explore possible sources of statistical heterogeneity in the EXACOS-CV program (unpublished data), and fitted models adjusted and not adjusted for smoking, for the risk of decompensated heart failure and of all-cause death. No difference was found between HRs, and bias incurred by not being able to adjust for smoking was deemed low.

Beyond statistical heterogeneity in terms of risk magnitude, unmeasured confounding is unlikely to have led to falsely positive results. Indeed, the results of all EXACOS-CV studies are consistent in terms of direction of the risk and align with previously published research. In addition, a critical assessment of bias in each included study was performed and sources of concern for bias were about potential underestimates rather than overestimates of association. For this reason, bias is unlikely to have implications for the conclusions of the meta-analysis (supplementary table S2). Finally, statistical heterogeneity across individual studies needs to be acknowledged. Statistical heterogeneity was mostly observed in the early exposure period (1–7 days), and for the outcome of a decompensated heart failure, probably due to methodological differences (*e.g.*, varying levels of misclassification of outcome and exposure) and true clinical diversity (*e.g.*, differences in patterns of cardiovascular event diagnosis and management of exacerbations of COPD), or differences in populations.

Finally, adjustment for several clinical characteristics related to disease severity, *e.g.* lung function, diastolic/systolic dysfunction and pulmonology data, could not be performed due to unavailable or poorly captured data.

### Conclusions

This meta-analysis of the EXACOS-CV studies underscores the consistent and significant risks for cardiovascular events that can occur following both moderate and severe exacerbations, which even newly diagnosed individuals are faced with. Prevention of exacerbations should be a core target of COPD management with optimised maintenance therapy to reduce the risk of further cardiopulmonary events. Future studies are needed to evaluate the impact of pharmacological and non-pharmacological interventions on the risk of cardiopulmonary outcomes.

## Supplementary material

**Please note:** supplementary material is not edited by the Editorial Office, and is uploaded as it has been supplied by the author. Supplementary material 01091-2024.SUPPLEMENT

## References

[1] Global Initiative for Chronic Obstructive Lung Disease (GOLD). Pocket Guide to COPD Diagnosis, Management, and Prevention. 2023. https://goldcopd.org/wp-content/uploads/2023/03/POCKET-GUIDE-GOLD-2023-ver-1.2-17Feb2023_WMV.pdf

[2] Whittaker H, Rubino A, Müllerová H, et al. Frequency and severity of exacerbations of COPD associated with future risk of exacerbations and mortality: a UK routine health care data study. Int J Chron Obstruct Pulmon Dis 2022; 17: 427–437. doi:10.2147/COPD.S346591 35264849 PMC8901192

[3] Müllerova H, Agusti A, Erqou S, et al. Cardiovascular comorbidity in COPD: systematic literature review. Chest 2013; 144: 1163–1178. doi:10.1378/chest.12-2847 23722528

[4] Chen W, Thomas J, Sadatsafavi M, et al. Risk of cardiovascular comorbidity in patients with chronic obstructive pulmonary disease: a systematic review and meta-analysis. Lancet Respir Med 2015; 3: 631–639. doi:10.1016/S2213-2600(15)00241-6 26208998

[5] Hurst JR, Gale CP, Global Working Group on Cardiopulmonary Risk. MACE in COPD: addressing cardiopulmonary risk. Lancet Respir Med 2024; 12: 345–348. doi:10.1016/S2213-2600(24)00038-9 38437859

[6] Müllerová H, Marshall J, de Nigris E, et al. Association of COPD exacerbations and acute cardiovascular events: a systematic review and meta-analysis. Ther Adv Respir Dis 2022; 16: 17534666221113647. doi:10.1177/17534666221113647 35894441 PMC9340406

[7] Nordon C, Rhodes K, Quint JK, et al. EXAcerbations of COPD and their OutcomeS on CardioVascular diseases (EXACOS-CV) Programme: protocol of multicountry observational cohort studies. BMJ Open 2023; 13: e070022. doi:10.1136/bmjopen-2022-070022 PMC10151875 37185641

[8] Daniels K, Lanes S, Tave A, et al. Risk of death and cardiovascular events following an exacerbation of COPD: The EXACOS-CV US Study. Int J Chron Obstruct Pulmon Dis 2024; 19: 225–241. doi:10.2147/COPD.S438893 38259591 PMC10802125

[9] Hawkins NM, Nordon C, Rhodes K, et al. Heightened long-term cardiovascular risks after exacerbation of chronic obstructive pulmonary disease. Heart 2024; 110: 702–709. doi:10.1136/heartjnl-2023-323487 38182279 PMC11103306

[10] Graul EL, Nordon C, Rhodes K, et al. Temporal risk of non-fatal cardiovascular events post COPD exacerbation: a population-based study. Am J Respir Crit Care Med 2023; 209: 960–972. doi:10.1164/rccm.202307-1122OC PMC11531205 38127850

[11] Swart KMA, Baak BN, Lemmens L, et al. Risk of cardiovascular events after an exacerbation of chronic obstructive pulmonary disease: results from the EXACOS-CV cohort study using the PHARMO Data Network in the Netherlands. Respir Res 2023; 24: 293. doi:10.1186/s12931-023-02601-4 37990197 PMC10662240

[12] Vogelmeier CF, Rhodes K, Garbe E, et al. Elucidating the risk of cardiopulmonary consequences of an exacerbation of COPD: results of the EXACOS-CV study in Germany. BMJ Open Respir Res 2024; 11: e002153. 10.1136/bmjresp-2023-002153 PMC10982767 38555102

[13] Santos S, Manito N, Sánchez-Covisa J, et al. Risk of severe cardiovascular events following COPD exacerbations: results from the EXACOS-CV study in Spain. Rev Esp Cardiol (Engl Ed) 2025; 78: 138–150. doi:10.1016/j.rec.2024.06.003 38936468

[14] Calabria S, Ronconi G, Dondi L, et al. Cardiovascular events after exacerbations of chronic obstructive pulmonary disease: results from the EXAcerbations of COPD and their OutcomeS in CardioVascular diseases study in Italy. Eur J Intern Med 2024; 127: 97–104. doi:10.1016/j.ejim.2024.04.021 38729787

[15] Higgins JPT, Morgan RL, Rooney AA, et al. A tool to assess risk of bias in non-randomized follow-up studies of exposure effects (ROBINS-E). Environ Int 2024; 186: 108602. doi:10.1016/j.envint.2024.108602 38555664 PMC11098530

[16] DerSimonian R, Laird N. Meta-analysis in clinical trials. Control Clin Trials 1986; 7: 177–188. doi:10.1016/0197-2456(86)90046-2 3802833

[17] Jackson D. Confidence intervals for the between-study variance in random effects meta-analysis using generalised Cochran heterogeneity statistics. Res Synth Methods 2013; 4: 220–229. doi:10.1002/jrsm.1081 26053842

[18] Higgins JPT, Thompson SG. Quantifying heterogeneity in a meta-analysis. Stat Med 2002; 21: 1539–1558. doi:10.1002/sim.1186 12111919

[19] Rücker G, Schwarzer G, Carpenter JR, et al. Undue reliance on I(2) in assessing heterogeneity may mislead. BMC Med Res Methodol 2008; 8: 79. doi:10.1186/1471-2288-8-79 19036172 PMC2648991

[20] Balduzzi S, Rücker G, Schwarzer G. How to perform a meta-analysis with R: a practical tutorial. Evid Based Ment Health 2019; 22: 153–160. doi:10.1136/ebmental-2019-300117 31563865 PMC10231495

[21] Brassington K, Selemidis S, Bozinovski S, et al. Chronic obstructive pulmonary disease and atherosclerosis: common mechanisms and novel therapeutics. Clin Sci (Lond) 2022; 136: 405–423. doi:10.1042/CS20210835 35319068 PMC8968302

[22] Simons S. Temporal dynamics of cardiovascular risk in patients with chronic obstructive pulmonary disease during stable disease and exacerbations: review of the mechanisms and implications. Int J Chron Obstruct Pulmon Dis 2024; 19: 2259–2271. doi:10.2147/COPD.S466280 39411574 PMC11474009

[23] Aaron SD, Vandemheen KL, Whitmore GA, et al. Early diagnosis and treatment of COPD and asthma: a randomized, controlled trial. N Engl J Med 2024; 390: 2061–2073. doi:10.1056/NEJMoa2401389 38767248

[24] Pullen R, Miravitlles M, Sharma A, et al. CONQUEST quality standards: for the collaboration on quality improvement initiative for achieving excellence in standards of COPD care. Int J Chron Obstruct Pulmon Dis 2021; 16: 2301–2322. doi:10.2147/COPD.S313498 34413639 PMC8370848

[25] Licskai C, Hussey A, Rowley V, et al. Quantifying sustained health system benefits of primary care-based integrated disease management for COPD: a 6-year interrupted time series study. Thorax 2024; 79: 725–734. doi:10.1136/thorax-2023-221211 38889973 PMC11287652

[26] Agusti A, Böhm M, Celli B, et al. GOLD COPD DOCUMENT 2023: a brief update for practicing cardiologists. Clin Res Cardiol 2024; 113: 195–204. doi:10.1007/s00392-023-02217-0 37233751 PMC10215047

[27] de Miguel-Díez J, Núñez Villota J, Santos Pérez S, et al. Multidisciplinary management of patients with chronic obstructive pulmonary disease and cardiovascular disease. Arch Bronconeumol 2024; 60: 226–237. doi:10.1016/j.arbres.2024.01.013 38383272

[28] Polman R, Hurst JR, Uysal OF, et al. Cardiovascular disease and risk in COPD: a state of the art review. Expert Rev Cardiovasc Ther 2024; 22: 177–191. doi:10.1080/14779072.2024.2333786 38529639

[29] Goto T, Shimada YJ, Faridi MK, et al. Incidence of acute cardiovascular event after acute exacerbation of COPD. J Gen Intern Med 2018; 33: 1461–1468. doi:10.1007/s11606-018-4518-3 29948806 PMC6108996

[30] Donaldson GC, Hurst JR, Smith CJ, et al. Increased risk of myocardial infarction and stroke following exacerbation of COPD. Chest 2010; 137: 1091–1097. doi:10.1378/chest.09-2029 20022970

[31] Reilev M, Pottegård A, Lykkegaard J, et al. Increased risk of major adverse cardiac events following the onset of acute exacerbations of COPD. Respirology 2019; 24: 1183–1190. doi:10.1111/resp.13620 31222861

[32] Dransfield MT, Criner GJ, Halpin DMG, et al. Time-dependent risk of cardiovascular events following an exacerbation in patients with chronic obstructive pulmonary disease: post hoc analysis from the IMPACT trial. J Am Heart Assoc 2022; 11: e024350. doi:10.1161/JAHA.121.024350 36102236 PMC9683674

[33] Kunisaki KM, Dransfield MT, Anderson JA, et al. Exacerbations of chronic obstructive pulmonary disease and cardiac events. A post hoc cohort analysis from the SUMMIT randomized clinical trial. Am J Respir Crit Care Med 2018; 198: 51–57. doi:10.1164/rccm.201711-2239OC 29442524 PMC6913068

[34] Hou, D Li, Z Liu, M, et al. Exacerbations of chronic obstructive pulmonary disease and cardiovascular diseases (EXACOS-CV): a database study in China on mortality and severe cardiovascular events (abstract). Am J Respir Crit Care Med 2024; 209: A1866.

