# Supplementary material for: The sustained increase of cardiovascular risk following COPD exacerbations: meta-analyses of the EXACOS-CV studies
Source: ERJ Open Res. 2025 Jun 16;11(3):01091-2024. doi: 10.1183/23120541.01091-2024 (PMC12168178; doi:10.1183/23120541.01091-2024)
Supplement: Supplementary file 1 [file 01091-2024.SUPPLEMENT.pdf]

SUPPLEMENTARY MATERIAL 1

Summary of systemic literature review and methods used in *Müllerová H, et al. Association of COPD exacerbations and acute cardiovascular events: a systematic review and meta-analysis. Ther Adv Respir Dis. 2022 Jan-Dec;16:17534666221113647.*

Search strategy

The study was conducted in accordance with the Meta-analysis of Observational Studies in Epidemiology and the Preferred Reporting Items for Systematic Reviews and Meta-Analyses (PRISMA) guidelines for conducting and reporting systematic reviews. The study protocol was published via PROSPERO: International Prospective Register of Systematic Reviews (#CRD42020211055) and is available at the following link: https://www.crd.york.ac.uk/prospero/display_record.php?ID=CRD42020211055.

A systematic literature review of observational studies published since 2000 was conducted by searching literature databases (Medline and Embase). Studies were eligible if conducted in adults with COPD, exposed to either COPD exacerbation or acute CV events, with outcomes of acute CV events or COPD exacerbation reported. Studies were appraised for relevance, bias and quality. Meta-analyses, using random-effect models, were performed for each outcome of interest, thus providing a pooled relative risk (RR) and its 95% confidence interval.

Results

Eight studies were identified, of which seven were used for the meta-analyses examining the risk of CV events 1–3 months after an exacerbation compared with none. For stroke (six studies), RR was 1.68 (95% CI = 1.19–2.38). For AMI (six studies), RR was 2.43 (95% CI = 1.40–4.20). No studies exploring risk of exacerbation following an acute CV event were identified.

SUPPLEMENTARY MATERIAL 2

Methods from published papers

Germany

The WIG2 (Scientific Institute for Health Economics and Health System Research) is an anonymised healthcare claims database with longitudinal data from approximately 4.5 million individuals from different statutory health insurance funds in Germany. The database is representative of the German population, with respect to the distribution of age and sex. Information is collected in inpatient and outpatient care settings, and includes demographics, medical diagnoses, prescriptions and corresponding dispensations, and full billing information. Patients or the public were not involved in the design, or conduct, or reporting, or dissemination plans of our research.

Patients with a COPD diagnosis (International Classification of Disease, V.10, German Version, ICD-10 J44, J44.0 and J44.1) in the inpatient or outpatient setting documented between 1 January 2014 and 31 December 2018, and aged 40+ years, were included. The first COPD diagnosis defined cohort entry. Only patients with 2 years of data availability prior to cohort entry were included. In the absence of any COPD-related code prior to cohort entry, the patient was

considered to be newly diagnosed. Baseline was defined as the 24-month period preceding cohort entry.

Study follow-up began on the date of cohort entry and ended upon the first occurrence of the outcome of interest, or right censoring (leaving the database or 31 December 2019).

Statistical analysis

To gain insight into the temporality between exacerbations of COPD and severe CV events, a swimmer plot was used. Patient subgroups were defined based on exposure and outcome status during follow-up, according to having had 0, 1, 2 or 3 moderate or severe exacerbations, and according to having experienced the outcome of a first severe CV event, including all-cause death. The swimmer plot included a bar for each subgroup, showing the typical trajectory of a patient, from cohort entry through to the end of follow-up, via exposure to exacerbations, based on median time.

Crude incidence rates of outcomes per 100 person-years were calculated with corresponding exact Poisson 95% CIs, during unexposed and exposed periods of time following an exacerbation (all exacerbations, moderate only and severe only).

Time-dependent Cox proportional hazards models were used to estimate the association between (1) the rate of outcome (any severe CV event or death; any non-fatal severe CV event; and each type of event separately) and time following an exacerbation of any severity; (2) the rate of each composite outcome and time following a moderate, or a severe exacerbation separately; and (3) the rate of a severe CV event or death following a first, a second and a third exacerbation of COPD in newly diagnosed patients. The reference period consisted of all unexposed periods as defined above, as we hypothesised that the risk of a severe CV event or death would return to the level of risk observed prior to a first exacerbation after 365 days following exacerbation onset. Sensitivity analyses were conducted to confirm this hypothesis, using only the time prior to a first exacerbation (or censoring) as an unexposed period. All models were fitted with and without adjustment for all prespecified time-invariant and time-varying confounders. No adjustment was made for multiple comparisons, as the outcomes were deemed interdependant.

Canada

The study cohort was retrieved from Alberta Health's COPD chronic disease cohort,17 which contained sociodemographic and medical information on individuals living with COPD from multiple health administrative databases. The cohort selection period was 1 April 2014 to 31 March 2019. Individuals with a COPD diagnosis in the outpatient or inpatient setting before this period were considered as prevalent and assigned a cohort entry date of 1 April 2014. Individuals diagnosed after 1 April 2014 were considered newly diagnosed (incident) and assigned the date when they first met the COPD diagnosis algorithm as a cohort entry date. For inclusion in the study, individuals were required to be ≥40 years old at cohort entry, residents of Alberta in the fiscal year containing the cohort entry date, have data available for at least 24 months pre-cohort entry and have no diagnosis of COPD related to alpha-1 antitrypsin deficiency. Study follow-up started at cohort entry and ended at (1) the first occurrence of the outcome of interest or (2) censoring (loss to follow-up due to moving out of Alberta, or administrative censoring on 31 March 2020).

Data analysis

Baseline characteristics of patients were summarised using means, SD and frequency distributions. The first of each outcome of interest and causes of death were summarised using frequency and percent distributions. Crude incidence rates of the composite and individual outcomes during the

unexposed and exposed subperiods were expressed per 100 person-years of follow-up; 95% CIs were calculated using the exact Poisson method.

Time-dependent Cox regression models allowing for repeated exposure were used to compare the risk of outcome in the periods following an exacerbation against the reference period. Several models were fitted to estimate first the risk of the composite outcome in time following (1) an exacerbation of any severity and (2) a moderate or a severe exacerbation counted separately; and second, the risk of each individual outcome following an exacerbation of any severity. In these latter analyses, competing non-fatal events were handled as time-varying confounders and death was a censoring event. In a last model using the subpopulation of incident patients, the exposed subperiods were categorised as a first, a second or a third+exacerbation. No multiple-testing correction was applied. All models were fitted with and without adjustment for pre-specified confounders selected *a priori* based on the literature and clinical expertise. Those included time-invariant covariates (sex, comorbidities, cohort entry year, urban/rural residence and neighbourhood income quintile) and time-varying covariates (cardiac/metabolic agents, COPD medication use, general practitioner visits, number of exacerbations, winter season and time-varying age).

The NL

This retrospective cohort study used data from the PHARMO Data Network from the Netherlands. The source population included people who were registered as patients with participating GP's (in total 20% of the Dutch population) and whose GP records could be linked to hospital and out-patient pharmacy records, irrespective of whether they were hospitalized or received medication between 1 January 2014 and 31 December 2018 (45% of the GP population). Individuals aged $\geq$ 40 years, with an incident, GP-reported diagnosis of COPD (International Classification of Primary Care (ICPC) R95 or R91.01) in this period were selected. The entire available lookback period was used to confirm the incident nature of COPD diagnosis. The date of COPD diagnosis was cohort entry date (CED). A medical diagnosis code for COPD diagnosis was required to be confirmed by at least one of the following additional variables: a record of spirometry measurement, a hospitalisation with a discharge diagnosis for COPD or exacerbation of COPD (International Classification of Disease (ICD)-10 J44) or GP-reported Global Initiative For Chronic Obstructive Lung Disease (GOLD) classification recorded in 3 years prior, or 3 years following the CED. Patients were included in the study population if they had at least 12 months of data available before CED. Patients with alpha-1 antitrypsin deficiency (ICD-10 E88.0) were excluded.

Statistical analyses

Baseline characteristics of the study population were described overall, and separately in patients who have, or have not exacerbated during study follow-up. Crude incidence rates (IR) of each outcome (composite, non-fatal CV events only, and individual categories) per 100 person-years were obtained, together with the corresponding 95% exact Poisson confidence intervals (CI).

Time-dependent Cox proportional hazards models were used with binary indicators of each exposed time period as time-dependent covariates, and the unexposed period as the reference. Separate models were fitted for (1) the association between exposure time periods following a moderate or severe exacerbation and each endpoint of interest, (2) for the separate association with moderate and severe exacerbations, and (3) for the association with a first, second and third exacerbation. All models were fitted with and without adjustment for all pre-specified fixed and time-varying confounders. For the analyses that examined the first, second and third exacerbation, exacerbations after the third were not evaluated. Analyses for the time to first severe CV event of

each specific category, or all-cause death, were adjusted for the other "competing" CV events of interest, as time-varying confounders. All analyses of time to a first severe CV event were censored when patients died without experiencing the outcome of interest.

Spain

The cohort entry date was the first identified COPD diagnosis, which was ascertained in the database by either: ≥ 2 outpatient visits with COPD diagnosis codes, or ≥ 1 outpatient visit with a COPD diagnosis code and indication for spirometry measurements being performed (3 years before or following the COPD code date), or ≥ 1 inpatient COPD diagnosis code. If ≥ 2 diagnosis dates were identified through inpatient or outpatient visits, the earliest defined the cohort entry date. Eligible patients were aged ≥ 40 years at cohort entry with a COPD diagnosis between 1 January 2014 and 31 December 2018. This age inclusion criterion is considered a standard method for improving the specificity of an algorithm to detect individuals with COPD when using secondary data. Patients were excluded if they had alpha-1 antitrypsin deficiency (International Classification of Diseases, Ninth Revision [ICD-9 273.8 and 273.9] or Tenth Revision [ICD-10 E88.01]). Codes for COPD diagnosis and related exacerbations, as defined in the Spanish Ministry of Health coding manual. The incident cohort was a subgroup of the overall cohort presumed to be newly diagnosed with COPD, as defined by having no COPD diagnosis code for 24 months before cohort entry.

Italy

To conduct this observational retrospective longitudinal cohort study, patients with COPD and aged 45 years or older were identified between January 1st 2015 and December 31st 2018 (identification period). The first criterion which identified a patient with COPD was defined as the index date. A minimum 24-month lookback period was required prior to index date.

Patients aged ≥45 were identified as having COPD by at least one of the following criteria: criterion (A) at least one hospitalization with a primary or secondary diagnosis of COPD in the hospital discharge forms (ICD-9CM codes: 491.x, 492.x, 496); criterion (B) a disease waiver claims for COPD (057); criterion (C) at least 4 dispensations of drugs for obstructive airway diseases (ATC code: R03) within a same 12-month period.

Patients with at least one day of follow-up after the index date were included. Patients with Alpha-1-antitrypsin deficiency (ICD-9-CM code 273.4) and, among those meeting only the inclusion criterion C, patients with asthma (identifying criteria are listed in supplementary Table 1) during the identification period were excluded.

Patients were followed from the index date until the first occurrence of a severe CV event or right censoring due to reaching the end of the study period on 31st December 2019 or the loss to follow-up (i.e., death or exit from the demographics database, for example due to the transfer to a Health Authority not included in the ReS database or another Country). The year 2020 was not considered for inclusion in the follow-up period, because the COVID-19 pandemic could have modified the risk of exacerbation and CV events and had an impact on the healthcare of patients.

Both newly diagnosed (defined as the absence of COPD criteria within the 24-month look-back period) and previously diagnosed patients with COPD were included and will henceforth be referred to as incident and prevalent patients, respectively.

Statistical analyses

Continuous variables were described using the mean, standard deviation (SD), median, inter-quartile range (IQR), min and max values. Dichotomous variables were described using n (%) of

each category. The number of patients experiencing a CV event during follow-up was reported, overall and by CV event (non-fatal or fatal).

Crude incidence rates (IR) of any type of CV event per 100 person-year were obtained for unexposed and exposed time periods and reported with 95 % exact Poisson confidence intervals (CI). Crude IRs were calculated as the number of first CV events divided by the total person years of follow-up. To obtain the IR during exposed time, total person years of exposed follow-up was calculated as the sum of all follow-up years that were within 1–365 days following a moderate or severe exacerbation. To obtain the IR during unexposed time, the total person years of unexposed time was calculated as the sum of all follow-up years that were outside 1–365 days following a moderate or severe exacerbation. Kaplan Meier estimates of the cumulative incidence of a CV event (i.e., any, acute coronary syndrome event, HF, cerebral ischemia, and cardiac arrhythmia) were obtained and plotted over time following the index date.

Cox proportional hazard regression was used to model time to first severe CV event in terms of time-dependent binary indicators of each exposure time period (i.e., 1–7, 8–14, 15–30, 31–180 and 181–365 days following the onset of a moderate or severe exacerbation). A separate Cox model was fitted for each outcome of interest (i.e., any type, and each type of CV event). Models estimated hazard ratios (HR) for the risk of a first severe CV event associated with each exposure sub-time period, compared to unexposed periods (i.e., all follow-up times outside of 1–365 days following an exacerbation). All models were fitted with and without adjustment for all pre-specified confounders.

Japan

Not published

The UK

We conducted an open cohort study including people with both new diagnoses of and long-standing diagnoses of COPD. People were eligible to be included in the cohort if they 1) were aged 40 years or older; 2) had a COPD diagnosis, the definition of which is based on a validated algorithm (86.5% predictive positive value); 3) were eligible for linkage with HES and Office for National Statistics data; 4) had smoking histories (i.e., current or ex-smokers); 5) had continuous GP practice registration with data of acceptable quality in the year before the start of follow-up; and 6) had data recorded after January 1, 2014. The index date in the exacerbation group (exposed) was the date of the first exacerbation after meeting eligibility criteria. For the nonexacerbating group (unexposed), the index date was the latest of COPD diagnosis date, 40th birthday, at least one year of continuous data of acceptable quality, and January 1, 2014. Follow-up was from the index date until December 31, 2019, or earlier if patients died or transferred GP practice. Minimum follow-up was one day.

Covariates

We had a “core” set of covariates for which we adjusted models, including age, sex, Index of Multiple Deprivation, smoking history, body mass index, type 2 diabetes, current asthma, depression, anxiety, hypertension, venous thromboembolism, COPD medication, and CVD medication. In sensitivity analyses only, in addition to the core set of covariates, we adjusted for a “sensitivity” set of covariates, including Medical Research Council (MRC) dyspnea score, Global Initiative for Chronic Obstructive Lung Disease (GOLD) grade, and chronic kidney disease, because of missing data (see Table E8.3 for extended covariate definitions). We chose covariates for models on the basis of 1) clinical expertise, 2) previous literature demonstrating relationships

between selected covariates and cardiovascular events, and 3) previous research we have done on the COPD–CVD relationship using similar covariates (as appropriate).

Statistical Analysis

Baseline characteristics were described overall and by exacerbation status. Absolute risk (percentage) and crude incidence rates were calculated across follow-up for patients with exacerbations (of any severity and by severity) and for patients without exacerbations.

Prespecification of subperiod hazards

We anticipated the effect of exacerbation on cardiovascular events would not be constant over time (i.e., violating the Cox proportional-hazards assumption). We visualized the instantaneous hazard of the event over time and assessed event rates across time to prespecify the following six subperiods, focusing within the first year: 1–14 days, 14–30 days, 30 days to 3 months, 3–9 months, 9 months to 1 year, and 1 year to the end of follow-up (see Appendix E8 for extended methodology).

Survival analyses

Cox regression was used to investigate time from the index date to the first composite nonfatal cardiovascular event, comparing patients with and those without exacerbations, adjusted for all "core set" covariates. Before running final multivariable models, we examined covariates for potential collinearity, and no collinearity was found (see Table E8.4). Models were fitted with and without stratification of time (according to the six prespecified subperiods). We applied Bonferroni corrections to the significance level for time-stratified models (see Table E8.5 and see Appendix E8 for extended methodology).

The US

The study population included newly diagnosed COPD patients defined as having a primary diagnosis code for COPD at an outpatient, inpatient, or emergency department (ED) visit between January 1, 2012, and December 30, 2019. The date of the first COPD diagnosis code between 2012 and 2019 was considered the incident diagnosis date and patients were excluded if they had any prior COPD diagnoses within 1 year or more. Patients were at least 40 years old on the date of their incident COPD diagnosis and had at least 12 months of continuous health plan enrollment prior to this date (Figure 1). The study period started January 1, 2006, and ended December 31, 2019, to avoid Coronavirus disease of 2019 (COVID-19) pandemic-related changes in health care utilization, patient behavior, and diagnosis misclassification between COPD exacerbations and COVID-19 infections. The baseline period was defined as all continuous enrollment time before the index date, which was the exacerbation date or, for comparators, a proxy date. Patients were excluded from study cohorts if they had an acute CV event within 6 months of their index date as a washout period to ensure that any CV diagnoses seen during follow-up were incident CV events. In the primary analysis, patients were followed starting on the index date until an outcome event, death (if not the outcome), disenrollment from their health plan, a subsequent AECOPD, or end of the study period. If someone in the exacerbation group had a second exacerbation, they were censored from the primary analysis and were then eligible for inclusion in a separate analysis of patients who had two exacerbations compared with patients who had one exacerbation.

Statistical analysis

Cox proportional hazards regression models were used to estimate average and time-stratified hazard ratios (HRs) of mortality and acute CV events comparing patients experiencing a first exacerbation (1-AECOPD) to patients with no prior exacerbation (0-AECOPD comparators). For

all models, covariates included age, sex, neighborhood (ie, census block-group) urbanicity, neighborhood socioeconomic status (SES), history of CV and COPD medication dispensings, duration of COPD, and COPD and CV comorbidities. Covariates were selected based on literature showing their association with the outcomes under study and were identified using National Drug Codes, ICD-9-CM and ICD-10-CM diagnosis codes, or from the American Community Survey. HRs of all-cause death and of acute CV event (of any type) following a first exacerbation, compared to no prior exacerbation, were estimated overall and separately by duration of follow-up. HRs of each type of acute CV event in the 30-day period following a first exacerbation compared to no prior exacerbation were also estimated.

The HRs of all-cause death and of acute CV events (of any type) following a first exacerbation of any severity (compared to no prior exacerbation) were analyzed by neighborhood urbanicity, SES, third-party payor type, number of lower respiratory infections one-year prior to index, number of short-acting beta 2-agonists (SABA) dispensings one-year prior to index, and by history of acute CV events.

HRs of all-cause death and of acute CV events (of any type) in the 30-days following a first moderate or a first severe AECOPD, compared to no prior AECOPD, were estimated. We also estimated the 30-day HRs of all-cause mortality and of acute CV events following a second AECOPD (vs one AECOPD) and following a third AECOPD (vs two AECOPD). Models for this set of analyses were adjusted for time since the previous exacerbation (eg, time since the first AECOPD in comparisons of the 2-AECOPD cohort to 1-AECOPD comparator cohort) and severity of the exacerbation, in addition to the covariates listed above.

SUPPLEMENTARY TABLE S1: Study design								
	**Germany**	**Canada**	**The NL**	**Spain**	**Italy**	**Japan**	**The UK**	**The US**
**Inclusion period**	1 Jan 2014 to 31 Dec 2018	1 Apr 2014 to 31 Mar 2019	1 Jan 2014 to 31 Dec 2018	1 Jan 2014 to 31 Dec 2019	1 Jan 2015 to 31 Dec 2018	1 Jan 2015 to 31 Dec 2018	1 Jan 2014 to 31 Dec 2019	1 Jan 2012 to 30 Dec 2019
**Cohort entry or index date**	The first identified COPD diagnosis during the inclusion period	The first identified COPD diagnosis during the inclusion period	The first identified COPD diagnosis during the inclusion period	≥2 outpatient visits with COPD diagnosis the earliest date defined the index date<br>o Or, in case only one outpatient visit COPD diagnosis, the presence of a spirometry measurement recorded in 3 years prior or 3 years following the date of the COPD diagnosis - the date of the outpatient visit defined index date;<br>o Or ≥1 inpatient COPD diagnosis	The first date when either of criteria A, B or C for COPD diagnosis (see below) was met was the index date	The first identified COPD diagnosis during the inclusion period	The index date in the exacerbation group (exposed) was the date of the first exacerbation after meeting eligibility criteria. For the nonexacerbating group (unexposed), the index date was the latest of COPD diagnosis date, 40th birthday, at least one year of continuous data of acceptable quality, and January 1, 2014.	Index date for AECOPD patients: the date of a subject's first AECOPD. Index date for comparator patients: An assigned date for all patients in the study population based on the distribution of time from incident COPD diagnosis to first AECOPD in the group having at least one AECOPD
**Newly diagnosed patients and prevalent patients**	In the absence of any COPD-related code prior to cohort entry, the patient was considered to be newly diagnosed.	The cohort selection period was 1 April 2014 to 31 March 2019. Individuals with a COPD diagnosis before this period were considered as prevalent and	Individuals aged ≥ 40 years, with an incident, GP-reported diagnosis of COPD in this period were selected. The entire available lookback period	The incident cohort was a subgroup of the overall cohort presumed to be newly diagnosed with COPD, as defined by having no COPD diagnosis code	Both newly diagnosed (defined as the absence of COPD criteria within the 24-month look-back period) and previously diagnosed	NA	NA	Defined as having a primary diagnosis code for COPD at an outpatient, inpatient, or emergency department (ED) visit between January 1, 2012,

		assigned a cohort entry date of 1 April 2014. Individuals diagnosed after 1 April 2014 were considered newly diagnosed.	was used to confirm the incident nature of COPD diagnosis.	for 24 months before cohort entry	patients with COPD were included and will henceforth be referred to as incident and prevalent patients, respectively.			and December 30, 2019.
**COPD diagnosis**	At least two confirmed outpatient ICD-10-GM diagnoses for COPD (as primary codes) in two different quarters of a year and/or at least one inpatient ICD-10-GM diagnosis for COPD (primary or secondary codes) within the inclusion period; In case ≥2 diagnosis dates were identified during the inclusion period, the earliest one in the screening period defined the index date	≥2 outpatient visits (physician claims) in the primary position on separate days and within 2 years (2nd visit as index date). One inpatient admission in any position (discharge date as index date)	A diagnosis of COPD identified using (a) the ICPC coding system, or (b) free text description of the diagnosis as provided by GPs and (a) a GP-reported GOLD classification (b) a spirometry measurement, regardless of result recorded in 3 years prior, or 3 years following the date of the first identified diagnosis of COPD	Two criteria were used:<br>- diagnosis code(s) for COPD (as primary or secondary diagnosis) in the BIG-PAC® database<br>- physician-reported spirometry measurement, or spirometry claims and recorded in 3 years prior or 3 years following the date of the first identified diagnosis of COPD	Patients with a diagnosis of COPD, defined as at least one of the following criteria<br>o Criteria A: At least one hospitalization mentioning COPD (ICD-9) in a primary or secondary discharge code;<br>o Criteria B: a disease waiver claim for COPD;<br>o Criteria C: at least four dispensations of COPD-related drugs within a same 12-month period; when only Criteria C was used, patients with asthma during the screening period will not be considered as	At least two diagnosis code for COPD in the outpatient setting, or ≥1 diagnosis discharge code for COPD in the inpatient setting identified. In case of several codes, the date of the first one will be used as cohort entry date (CED)	1. Had data recorded in CPRD Aurum from 1 January 2004 onwards;<br>2. Had a diagnosis of COPD using validated codes;<br>3. Were aged ≥40 years old at (the first) COPD diagnosis;<br>4. Were current or ex-smokers;<br>5. Were registered at a GP practice that was UTS	At least one primary diagnosis of COPD between 1 January 2012 and 30 December 2019, made at an outpatient, emergency department, or inpatient visit

					COPD patients			
**Inclusion/exclusion criteria**	Only patients with 2 years of data availability prior to cohort entry were included	For inclusion in the study, individuals were required to be ≥40 years old at cohort entry, residents of Alberta in the fiscal year containing the cohort entry date, have data available for at least 24 months pre-cohort entry and have no diagnosis of COPD related to alpha-1 antitrypsin deficiency.	Patients were included in the study population if they had at least 12 months of data available before CED. Patients with alpha-1 antitrypsin deficiency (ICD-10 E88.0) were excluded.	Eligible patients were aged ≥ 40 years at cohort entry with a COPD diagnosis between 1 January 2014 and 31 December 2018. This age inclusion criterion is considered a standard method for improving the specificity of an algorithm to detect individuals with COPD when using secondary data. Patients were excluded if they had alpha-1 antitrypsin deficiency (International Classification of Diseases, Ninth Revision [ICD-9 273.8 and 273.9] or Tenth Revision [ICD-10 E88.01]).	To conduct this observational retrospective longitudinal cohort study, patients with COPD and aged 45 years or older were identified between January 1st 2015 and December 31st 2018 (identification period). The first criterion which identified a patient with COPD was defined as the index date (supplementary figure 2). A minimum 24-month lookback period was required prior to index date.		People were eligible to be included in the cohort if they 1) were aged 40 years or older; 2) had a COPD diagnosis, the definition of which is based on a validated algorithm (86.5% predictive positive value); 3) were eligible for linkage with HES and Office for National Statistics data; 4) had smoking histories (i.e., current or ex-smokers); 5) had continuous GP practice registration with data of acceptable quality in the year before the start of follow-up; and 6) had data recorded after January 1, 2014.	Patients were at least 40 years old on the date of their incident COPD diagnosis and had at least 12 months of continuous health plan enrollment prior to this date
**Moderate exacerbation**	• a dispensation for a systemic corticosteroid (prednisone or	Physician outpatient visit (respiratory medicine,	• an outpatient visit to the GP for COPD, or for an exacerbation	An outpatient visit to the GP or a pulmonologist with a diagnosis	A dispensation (order or fill) of systemic corticosteroids	• an outpatient visit with a diagnosis code (any position) for	• an outpatient visit to the GP with a SNOMED-CT	An outpatient visit with a diagnosis of COPD

	prednisolone) with a dosage of >20 mg/day of prednisolone equivalent and for a duration ≤15 days<br>• an outpatient visit to the GP or a pulmonologist, or an internist, with a diagnosis code for COPD taking place in the same quarter of the year as the drug prescription	cardiologist, pulmonologist, general/family physician, internal medicine) with a diagnosis code for COPD<br>• and a new dispense of OCs within 5 days of the visit (before or after) for a duration of <15 days or of antibiotics for respiratory infections within 5 days of the visit (before or after) duration of ≤15 days	of COPD as identified using search terms (free text as provided by the GP)<br>• and a dispensation for an OC (prednisone or prednisolone) within the 5 days following the visit, for a duration of ≤15 days and ≥5 days	code for COPD and a dispensation for an OC (prednisone or prednisolone) and/or respiratory antibiotics (ATC codes J01AA and J0A1CA) within the 5 days following the visit, for a maximum duration of ≤15 days, and using integrated records available	and of respiratory antibiotics on the same day for a duration ≤15 days	COPD (J41–J44) or for acute bronchitis (J20–J22) and<br>• a dispensation claim for injectable or OC with a dose of ≥20 mg/day prednisolone equivalent within the 5 days of the visit and for a duration of ≤15 days; or of oral antibiotics within the 5 days of the visit and for a duration of ≤15 days	concept ID for 'lower respiratory tract infection' or 'COPD exacerbation'; or SNOMED-CT concept ID indicating acute respiratory symptoms<br>• and a prescription of antibiotics or of OCs for a duration of 5–14 days (not on the same day as an annual review visit, for example, prescribed as a rescue pack)	and, within 7 days of the visit, a medication dispensing of ≤30 days' supply of oral glucocorticoids or antibiotics, or administration of intravenous (IV) glucocorticoids dispensation
**Severe exacerbation**	an emergency department visit or hospitalisation of ≥1 night<br>• for an exacerbation of COPD as the main reason for being hospitalised (i.e. using the primary discharge code) or for an exacerbation that occurred during a	• an emergency department visit with a COPD diagnosis in any position<br>• or hospitalisation of ≥1 night with a 'most responsible diagnosis' (reason for admission) code or post-admission diagnosis (complication occurring during	• hospitalisation of ≥1 night<br>• for COPD as identified in the discharge code as the main reason for being hospitalised, or for an exacerbation that occurred during a hospital stay	• an emergency department visit and/or hospitalisation for which a bed is required • with a primary or secondary discharge diagnosis code of COPD (ICD-10 code J44), or COPD exacerbation (ICD-10 code J44.0 or J44.1)	• hospitalisation of ≥1 night<br>• for an exacerbation of COPD as the main reason for being hospitalised (i.e. using the primary discharge code) or for an exacerbation that occurred during a hospital stay (primary diagnosis of COPD or secondary	• hospitalisation of ≥1 night<br>• with a discharge code for 'COPD' (ICD-10 J41–J44) as the 'Disease name behind hospitalisation' or 'COPD with (acute) lower respiratory infection' (ICD-10 J44.0) or 'COPD with (acute) exacerbation'	• hospitalisation of ≥1 night<br>• with a discharge code indicating that the reason for the hospitalisation is an exacerbation of COPD (i.e. using the first codes on the list are 'lower respiratory tract infection', followed by 'COPD' or 'COPD'; this is following	a hospitalisation or emergency department visit with either a primary diagnosis of COPD, a primary diagnosis of respiratory failure, acute bronchitis, asthma exacerbation, bronchiectasis exacerbation, or pneumonia with a secondary

	hospital stay (primary diagnosis of COPD or secondary diagnosis of COPD and main diagnosis of related causes)	the hospital stay) for COPD			diagnosis of COPD and main diagnosis of related causes)	(ICD-10 J44.1) as ‘disease name, which was seen as a complication after hospitalisation’ or as ‘Main disease name’	previous validation in HES	diagnosis of COPD
**Exposure periods**	1-7 days; 8-14 days; 15-30 days; 31-180 days; 181-365 days	1-7 days; 8-14 days; 15-30 days; 31-180 days; 181-365 days; >365 days	1-7 days; 8-14 days; 15-30 days; 31-180 days; 181-365 days	1-7 days; 8-14 days; 15-30 days; 31-180 days; 181-365 days; >365 days	1-7 days; 8-14 days; 15-30 days; 31-180 days; 181-365 days	1-7 days; 8-14 days; 15-30 days; 31-180 days; 181-365 days	1-14 days; 15-30 days; 31-90 days; 91-270 days (3-9 months); 271-365 days (9-12 months)	1-30 days; 31-90 days; 91-180 days;181-365 days; 366-730 days (12 months-2 years); 731-1095 days (2-3 years); >1095 days (3 years or more)
**Confounders**	All models were fitted with and without adjustment for all prespecified time-invariant and time-varying confounders. Age at cohort entry, Gender Socio-economic status, Winter season (December-February) when an exacerbation occurs,Urban area of living, Smoking	All models were fitted with and without adjustment for pre-specified confounders selected a priori based on the literature and clinical expertise. Those included time-invariant covariates (sex, comorbidities, cohort entry year, urban/rural residence and neighbourhood income quintile) and time-varying covariates	Baseline and time-dependent covariates were pre-specified as potential confounders. Baseline covariates that were defined over 12-month lookback period preceding CED were age, sex, socio-economic status (low/middle/high /unknown), and obesity (ICPC T82, ICD-10 E66, or body mass index > 30	Models were fitted with and without adjustment for prespecified confounders. These included age, sex, cohort entry year, smoking status, alcohol use disorder, comorbidities, primary care general practitioner visits, number of prior exacerbations, and medication use, along with	All models were fitted with and without adjustment for all pre-specified confounders. Age, Sex, Diabetes mellitus type-2, Dyslipidaemias, Ischaemic heart diseases, Arterial hypertension, Heart failure, Pulmonary oedema, Pulmonary hypertension, Venous thromboembolism,		A “core” set of covariates for which we adjusted models, including age, sex, Index of Multiple Deprivation, smoking history, body mass index, type 2 diabetes, current asthma, depression, anxiety, hypertension, venous thromboembolism, COPD medication, and CVD medication	For all models, covariates included age, sex, neighborhood (ie, census block-group) urbanicity, neighborhood socioeconomic status (SES), history of CV and COPD medication dispensings, duration of COPD, and COPD and CV comorbidities.

	history, Alcohol use disorder, Obesity, Diabetes mellitus type-2, Any Disorders of lipoprotein metabolism and other lipidaemias, Ischaemic heart diseases, Hypertensive diseases, Heart failure, Pulmonary oedema, Pulmonary hypertension, Venous thromboemboli sm, Cerebrovascula r disease, Arrythmia, Current asthma, Chronic kidney disease, renal failure, Any severe mental illness, Anxiety disorder, At least one dispensation of cardiac or metabolic agent of each therapeutic subgroup, At	(cardiac/metaboli c agents, COPD medication use, general practitioner visits, number of exacerbations, winter season and time-varying age).	kg/m2). Baseline covariates that were defined based on the entire available history (hospital ICD-9 or ICD-10 diagnosis or GP-reported ICPC diagnoses) included diabetes mellitus type 2, disorders of lipoprotein metabolism and other lipidaemias, ischaemic heart diseases, hypertensive diseases, heart failure, pulmonary oedema, pulmonary hypertension, venous thromboembolis m, cerebrovascular disease, arrhythmia, asthma, chronic kidney disease or renal failure, mental illness and/or anxiety disorder. Time-dependent covariates were updated annually	cardiovascular risk factors such as diabetes, hypertensive disease, and prior HF, among others. Analyses of individual cardiovascular event types and all-cause death included competing cardiovascular events as time-dependent covariates.	Cerebrovascular disease, Arrythmia, Chronic kidney disease, Psychiatric disorders, At least one dispensation of cardiac or metabolic agent of each therapeutic subgroup in the past 12 months, At least one dispensation of COPD-related drug in the past 12 months, Year of cohort entry, Incident patients (vs. prevalent patients) at the index date, Number of exacerbations within 12 months before the index date			

	least one dispensation of COPD categorised into:<br>- LABA<br>- LAMA<br>- ICS<br>- SABASAMA<br>- Oral drug (theophylline or roflumilast), Number of GP visits within the last 12 months, Year of cohort entry, Incident, COPD (vs. Prevalent), Number of exacerbations since the beginning of the 24-month baseline period		from CED onward unless otherwise stated and included COPD-related variables (number of GP visits in the last 12 months, number of prior moderate or severe exacerbations, and comedication use in the last 12 months (at least one prescription for long-acting inhaled bronchodilators, inhaled corticosteroids and their combination, short-acting inhalers, roflumilast and/or slow-release theophylline, any cardiac medication, any metabolic medication. Smoking wasn't included due to a substantial amount (> 50% of patients) of missing data.					

Abbreviations: AECOPD: acute exacerbation of COPD; ATC: Anatomical Therapeutic Chemical; CED: cohort entry date; COPD: chronic obstructive pulmonary disease; GP: general practitioner; ICD-10: International Statistical Classification of Diseases-10; ICPC: International Classification of Primary Care; IV: intravenous; OC: oral corticosteroid; SNOMED-CT: Systemized Nomenclature of Medicine – Clinical Terms; UK: United Kingdom; US: United States; UTS: up-to-standard.

SUPPLEMENTARY TABLE 2. EXACOS-CV studies included in the meta-analysis were critically assessed for risk of bias, according to the seven domains within the ROBINS-E tool (10.1016/j.envint.2024.108602). Sources of concern are summarised in the Table.

	Germany	*Canada*	*The Netherlands*	*Spain*	*Italy*	*Japan*	*United Kingdom*	*United States*
Bias due to confounding	Uncontrolled confounding may play a role; smoking history is likely to have been under-coded.<br><br>Over-reporting of comorbidities may be possible in claims data due to reimbursement reasons.	Unmeasured confounding is a concern; smoking status, alcohol use disorder and obesity were deemed inadequately collected and not adjusted for.	Unmeasured confounding cannot be ruled out, due to ability to capture smoking status and exposure to air pollutants, among other factors.	Unmeasured confounding cannot be ruled out, due to ability to capture exposure to air pollutants, among other factors.	Unmeasured confounding cannot be ruled out, due to ability to capture smoking status, alcohol use disorder, obesity and exposure to air pollutants, among other factors.<br><br>Over-reporting of comorbidities may be possible in claims data due to reimbursement reasons.	Unmeasured confounding cannot be ruled out, due to ability to capture exposure to air pollutants, among other factors.	Confounders were not time-updated. Bias would mainly concern associations with the later post-exacerbation periods.<br><br>Unmeasured confounding is a concern; there was substantial missing data COPD-related variables and ethnicity.	Confounders were not time-updated. Bias would mainly concern the associations with longer-term outcome risks.<br><br>Unmeasured confounding is a concern; race/ethnicity, socioeconomic status, and smoking status were not available.
Bias arising from measurement of the exposure	Moderate exacerbations treated only with antibiotics would be missed, possibly leading to underestimates of association.	Moderate exacerbations of a lower managed outside of the outpatient setting would be missed, possibly leading to underestimates of association.	ER visits not resulting in hospitalisation would be missed leading to underestimates of association.	Exacerbations would be missed if not recorded in the usual hospital that collects data used in this study, potentially leading to underestimation of associations.	Moderate exacerbations recorded during outpatient visits to the GP were not identifiable, potentially leading to underestimates of association.<br><br>Exacerbations would be	Exacerbations would be missed if not recorded in the usual hospital that collects data used in this study, potentially leading to underestimation of associations.	Time-varying exposures were not included. Unexposed patients may have had an exacerbation during follow up that increased their risk of a CV event. This would have led to	Moderate exacerbations managed outside of the outpatient setting would be missed, possibly leading to underestimates of association.<br><br>Patients were censored upon a

					missed if not recorded in the usual hospital that collects data used in this study, potentially leading to underestimation of associations.		underestimates of association.	subsequent exacerbation, therefore CV events following post-baseline exposure would be missed, leading to underestimation of association.
Bias in selection of participants into the study (or analysis)	The algorithm used to identify COPD patients was not validated; spirometry was not captured in the database, so could not be used to improve specificity. Thus, there is a possibility that non-COPD patients were included; this may have led to underestimates of association.					Without spirometry results for patients, there may be some patients with a COPD diagnosis who do not have COPD.		The identification of patients with COPD was based on the presence of only one COPD code and did not use a validated algorithm.<br><br>During follow-up, 40% of patients in the comparator cohort did not have any other COPD code identified, thus making the diagnosis of COPD less reliable than in the AECOPD cohort and potentially leading to overestimation of association.

Bias due to post-exposure interventions	This was handled in the analysis through adjustment for medication use as a time-varying confounder. Potentially this could have led to over-adjustment since the effect of an AECOPD on CV risk may be mediated by medication use.						Not accounted for in the analysis. Adjustment was made for pre-index date medications, and it may be reasonable to assume changes over time would be minimal, given the duration of follow up.	Not accounted for in the analysis. Adjustment was made for pre-index date medications, and it may be reasonable to assume changes over time would be minimal, given the limited follow up in claims data.
Bias due to missing data	Data were generated for reimbursement purposes; it is possible that events were present but not reported.<br><br>It was not possible to distinguish between absence of health condition vs. missing record; absence of record was deemed absence of condition.	It was not possible to distinguish between absence of health condition vs. missing record; absence of record was deemed absence of condition.	Before linkage of the pharmacy, hospital and GP data, patients for whom crucial information needed for linkage is missing (date of birth, sex, GP) were removed.<br><br>The absence of a clinical event or medication was assumed to indicate the absence of that condition or medication.	It was not possible to distinguish between absence of health condition vs. missing record; absence of record was deemed absence of condition.	Data were generated for reimbursement purposes; it is possible that events were present but not reported.<br><br>It was not possible to distinguish between absence of health condition vs. missing record; absence of record was deemed absence of condition.	Only in-hospital deaths were observed, leading to underestimation of associations between AECOPD and risk of outcomes including death.<br><br>It was not possible to distinguish between absence of health condition vs. missing record; absence of record was deemed absence of condition.	For most variables, missing was assumed to be negative status (i.e., absence of condition). However diagnostic recording in the database is of high quality in terms of completeness.	Patients with a death date preceding their index date, and missing demographic data were excluded.<br><br>Data were generated for reimbursement purposes; it is possible that events were present but not reported.<br><br>It was not possible to distinguish between absence of health condition vs. missing

								record; absence of record was deemed absence of condition.
Bias in measurement of the outcome	Due to preferential coding of certain conditions for reimbursement reasons, there may be some level of misclassification when outcome events occurred simultaneously with other event types.<br><br>Exacerbations and decompensated heart failure have similar symptoms and may have been misdiagnosed. Bias concerns overestimation of associations in early exposed periods.	The occurrence of a severe CV event was approximated by the presence of hospital discharge codes. In patients hospitalized for a supposed moderate or severe AECOPD, it is possible that symptoms observed could be due to a CV event (e.g., symptoms of ACS or HF). Expected bias is minimal since diagnosis is coded by physicians upon discharge after correct diagnosis is typically confirmed.	Potential exposure-outcome association; the temporality between AECOPD and outcome events cannot be precisely retrieved from discharge diagnosis codes. The exact date of severe CV events that may have occurred during hospital stay could not be determined.<br><br>Exacerbations and decompensated heart failure have similar symptoms and may have been misdiagnosed. Bias concerns overestimation of associations in early exposed periods.	CV events would be missed if not recorded in the usual hospital that collects data used in this study, potentially leading to underestimation of associations.<br><br>Exacerbations and decompensated heart failure have similar symptoms and may have been misdiagnosed. Bias concerns overestimation of associations in early exposed periods.	CV events would be missed if not recorded in the usual hospital that collects data used in this study, potentially leading to underestimation of associations.<br><br>Exacerbations and decompensated heart failure have similar symptoms and may have been misdiagnosed. Bias concerns overestimation of associations in early exposed periods.<br><br>Severe arrhythmias that occurred during a hospital stay were not measurable, thus underestimating the association.	Exacerbations and decompensated heart failure have similar symptoms and may have been misdiagnosed. Bias concerns overestimation of associations in early exposed periods.	CV events occurring post-exacerbation may have been incorrectly assigned to unexposed time, due to not measuring post-baseline exacerbations. This may have led to underestimation of associations.<br><br>It is also possible that there is some misdiagnosis or delayed diagnosis or misclassification of CV and AECOPD events given the overlap in symptoms, and lack of CV investigations readily available in primary care.	Claims data were used; there may be some level of misclassification when outcome events occurred simultaneously with other event types.<br><br>Some acute CV events such as pulmonary embolism have similar symptoms to an AECOPD and could be misdiagnosed and therefore misclassified.

Bias in selection of the reported result	Not a concern – results were all reported in accordance with a pre-defined statistical analysis plan

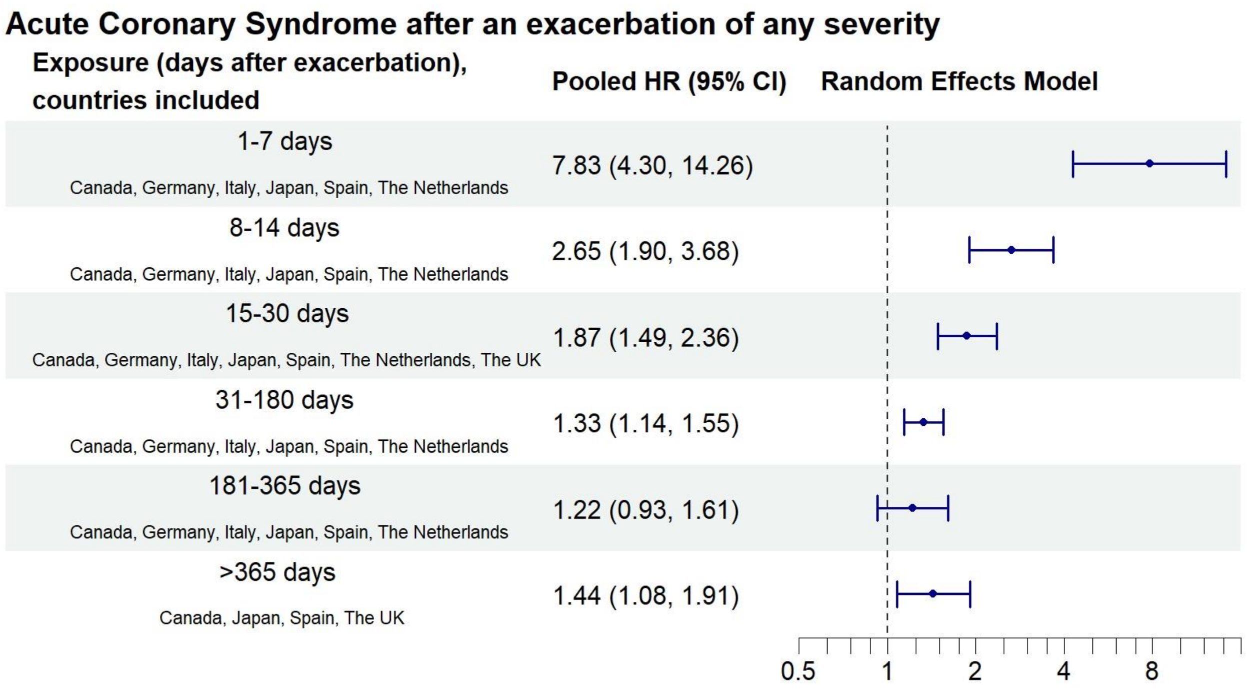

Supplementary Figure S1: Risk of acute coronary syndrome following an exacerbation of any severity, random effects meta-analysis of adjusted hazard ratios

Abbreviations: CI: confidence interval; CV: cardiovascular; HR: hazard ratio; UK: United Kingdom

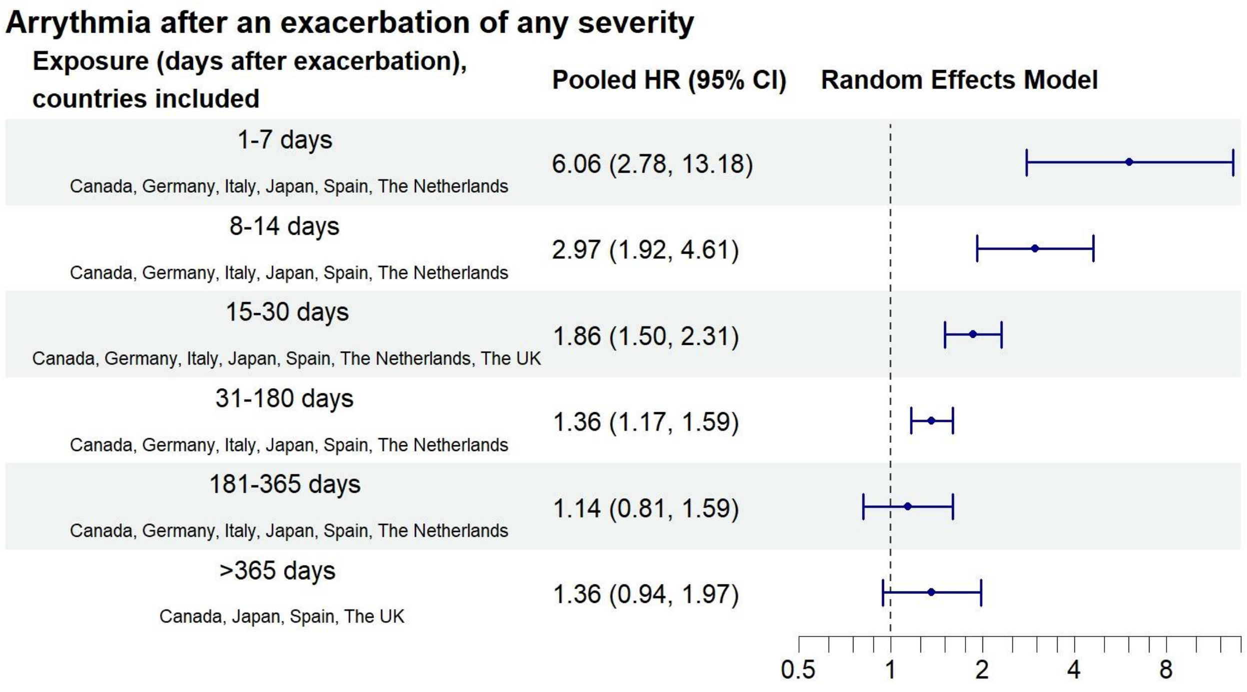

Supplementary Figure S2: Risk of arrythmia following an exacerbation of any severity, random effects meta-analysis of adjusted hazard ratios

Abbreviations: CI: confidence interval; CV: cardiovascular; HR: hazard ratio; UK: United Kingdom.

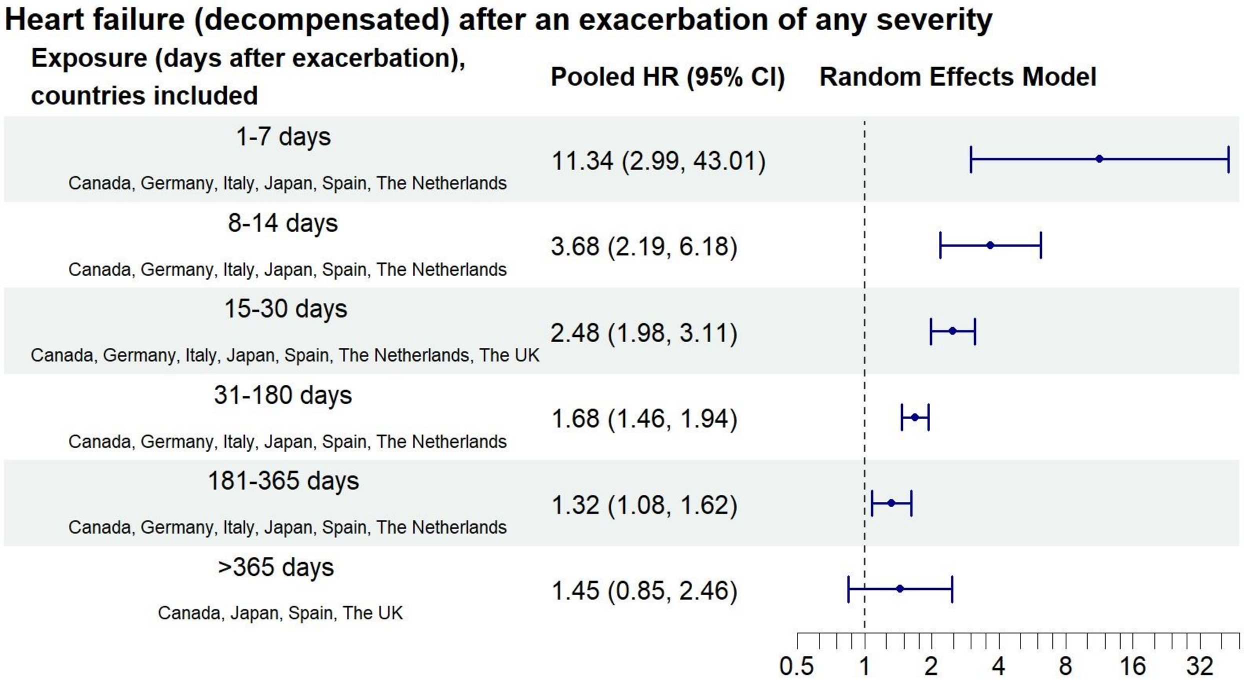

Supplementary Figure S3: Risk of heart failure following an exacerbation of any severity, random effects meta-analysis of adjusted hazard ratios

Abbreviations: CI: confidence interval; CV: cardiovascular; HR: hazard ratio; UK: United Kingdom.

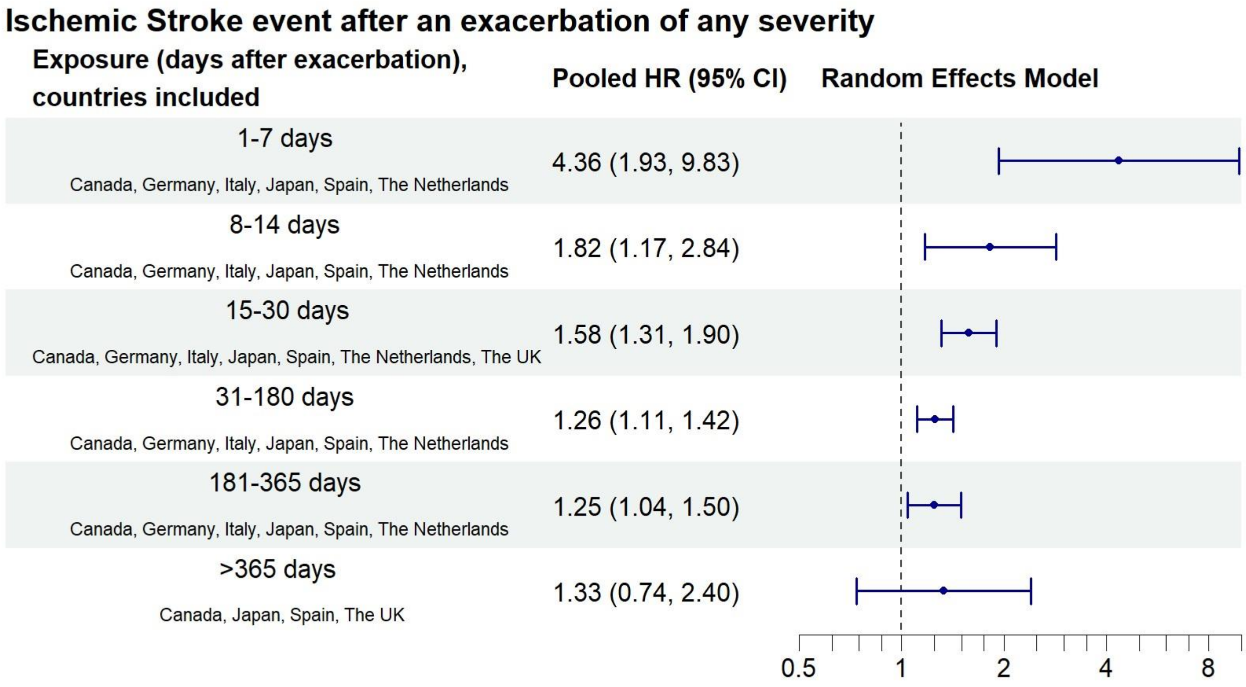

Supplementary Figure S4: Risk of ischaemic stroke following an exacerbation of any severity, random effects meta-analysis of adjusted hazard ratios

Abbreviations: CI: confidence interval; CV: cardiovascular; HR: hazard ratio; UK: United Kingdom.

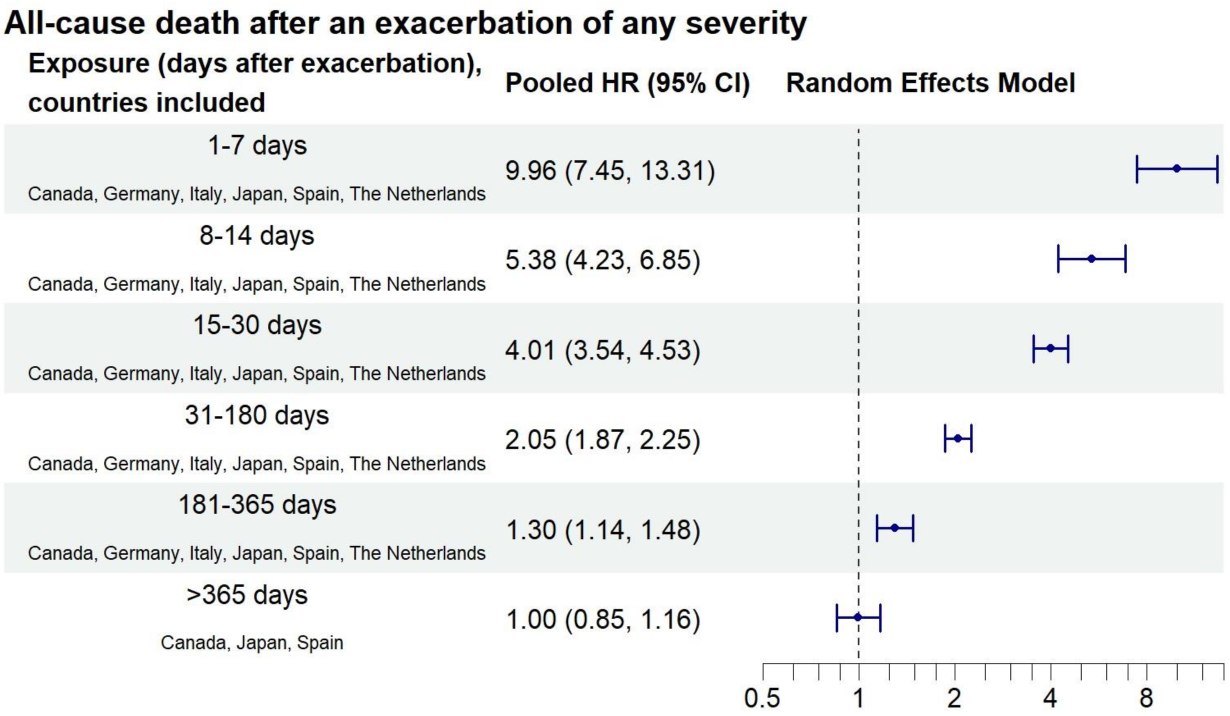

Supplementary Figure S5: Risk of all-cause death following an exacerbation of any severity, random effects meta-analysis of adjusted hazard ratios

Abbreviations: CI: confidence interval; CV: cardiovascular; HR: hazard ratio.

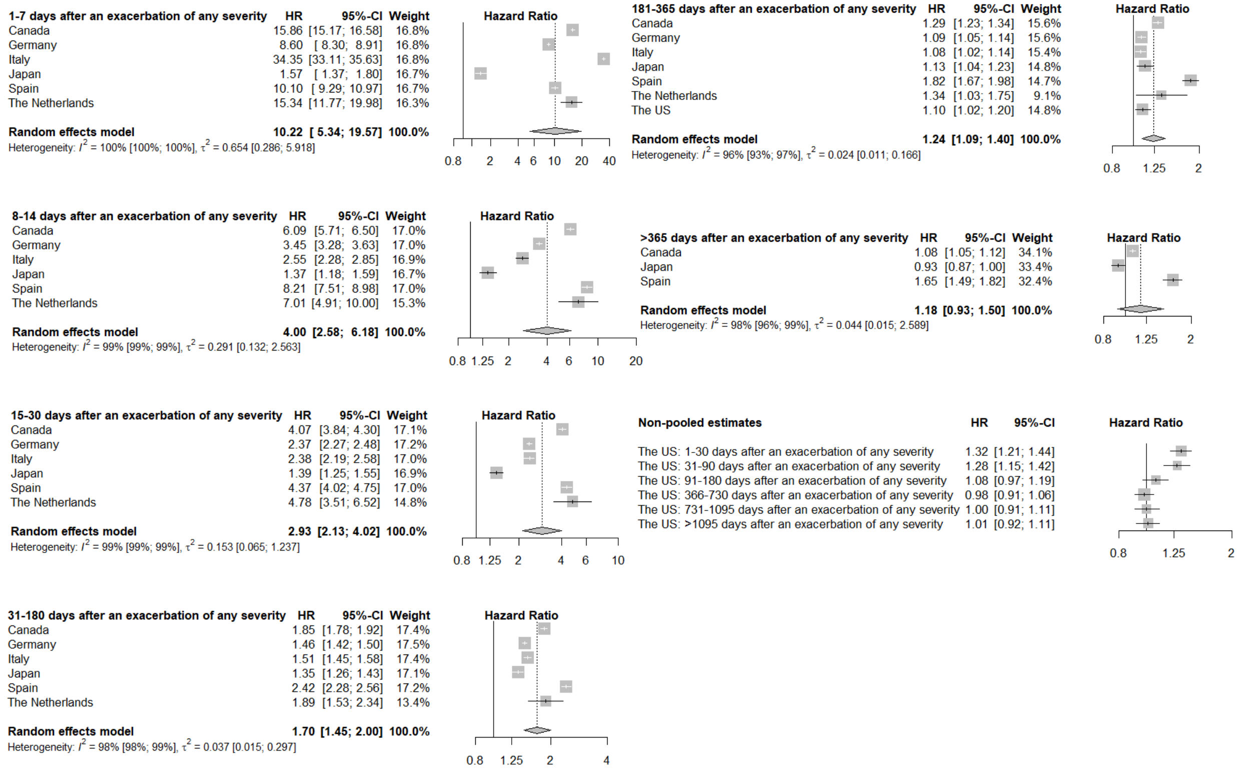

1-7 days after an exacerbation of any severity	HR	95%-CI	Weight
Canada	15.86	[15.17; 16.58]	16.8%
Germany	8.60	[8.30; 8.91]	16.8%
Italy	34.35	[33.11; 35.63]	16.8%
Japan	1.57	[1.37; 1.80]	16.7%
Spain	10.10	[9.29; 10.97]	16.7%
The Netherlands	15.34	[11.77; 19.98]	16.3%
**Random effects model**	**10.22**	**[5.34; 19.57]**	**100.0%**

Heterogeneity: I^2 = 100% [100%; 100%], τ^2 = 0.654 [0.286; 5.918]

8-14 days after an exacerbation of any severity	HR	95%-CI	Weight
Canada	6.09	[5.71; 6.50]	17.0%
Germany	3.45	[3.28; 3.63]	17.0%
Italy	2.55	[2.28; 2.85]	16.9%
Japan	1.37	[1.18; 1.59]	16.7%
Spain	8.21	[7.51; 8.98]	17.0%
The Netherlands	7.01	[4.91; 10.00]	15.3%
**Random effects model**	**4.00**	**[2.58; 6.18]**	**100.0%**

Heterogeneity: I^2 = 99% [99%; 99%], τ^2 = 0.291 [0.132; 2.563]

15-30 days after an exacerbation of any severity	HR	95%-CI	Weight
Canada	4.07	[3.84; 4.30]	17.1%
Germany	2.37	[2.27; 2.48]	17.2%
Italy	2.38	[2.19; 2.58]	17.0%
Japan	1.39	[1.25; 1.55]	16.9%
Spain	4.37	[4.02; 4.75]	17.0%
The Netherlands	4.78	[3.51; 6.52]	14.8%
**Random effects model**	**2.93**	**[2.13; 4.02]**	**100.0%**

Heterogeneity: I^2 = 99% [99%; 99%], τ^2 = 0.153 [0.065; 1.237]

31-180 days after an exacerbation of any severity	HR	95%-CI	Weight
Canada	1.85	[1.78; 1.92]	17.4%
Germany	1.46	[1.42; 1.50]	17.5%
Italy	1.51	[1.45; 1.58]	17.4%
Japan	1.35	[1.26; 1.43]	17.1%
Spain	2.42	[2.28; 2.56]	17.2%
The Netherlands	1.89	[1.53; 2.34]	13.4%
**Random effects model**	**1.70**	**[1.45; 2.00]**	**100.0%**

Heterogeneity: I^2 = 98% [98%; 99%], τ^2 = 0.037 [0.015; 0.297]

181-365 days after an exacerbation of any severity	HR	95%-CI	Weight
Canada	1.29	[1.23; 1.34]	15.6%
Germany	1.09	[1.05; 1.14]	15.6%
Italy	1.08	[1.02; 1.14]	15.4%
Japan	1.13	[1.04; 1.23]	14.8%
Spain	1.82	[1.67; 1.98]	14.7%
The Netherlands	1.34	[1.03; 1.75]	9.1%
The US	1.10	[1.02; 1.20]	14.8%
**Random effects model**	**1.24**	**[1.09; 1.40]**	**100.0%**

Heterogeneity: I^2 = 96% [93%; 97%], τ^2 = 0.024 [0.011; 0.166]

>365 days after an exacerbation of any severity	HR	95%-CI	Weight
Canada	1.08	[1.05; 1.12]	34.1%
Japan	0.93	[0.87; 1.00]	33.4%
Spain	1.65	[1.49; 1.82]	32.4%
**Random effects model**	**1.18**	**[0.93; 1.50]**	**100.0%**

Heterogeneity: I^2 = 98% [96%; 99%], τ^2 = 0.044 [0.015; 2.589]

Non-pooled estimates	HR	95%-CI
The US: 1-30 days after an exacerbation of any severity	1.32	[1.21; 1.44]
The US: 31-90 days after an exacerbation of any severity	1.28	[1.15; 1.42]
The US: 91-180 days after an exacerbation of any severity	1.08	[0.97; 1.19]
The US: 366-730 days after an exacerbation of any severity	0.98	[0.91; 1.06]
The US: 731-1095 days after an exacerbation of any severity	1.00	[0.91; 1.11]
The US: >1095 days after an exacerbation of any severity	1.01	[0.92; 1.11]

Supplementary Figure S6: Severe cardiovascular event including death after an exacerbation of any severity; statistical heterogeneity among study results

Higgins' and Thompson's I^2 statistic was computed to quantify the proportion of variation among estimated HRs that is due to between-study heterogeneity. The DerSimonian-Laird estimator [15] was used to quantify between-study variance τ^2, and the method of Jackson was used to compute the 95% CI for τ^2

Abbreviations: CI: confidence interval; CV: cardiovascular; HR: hazard ratio; US: United States.

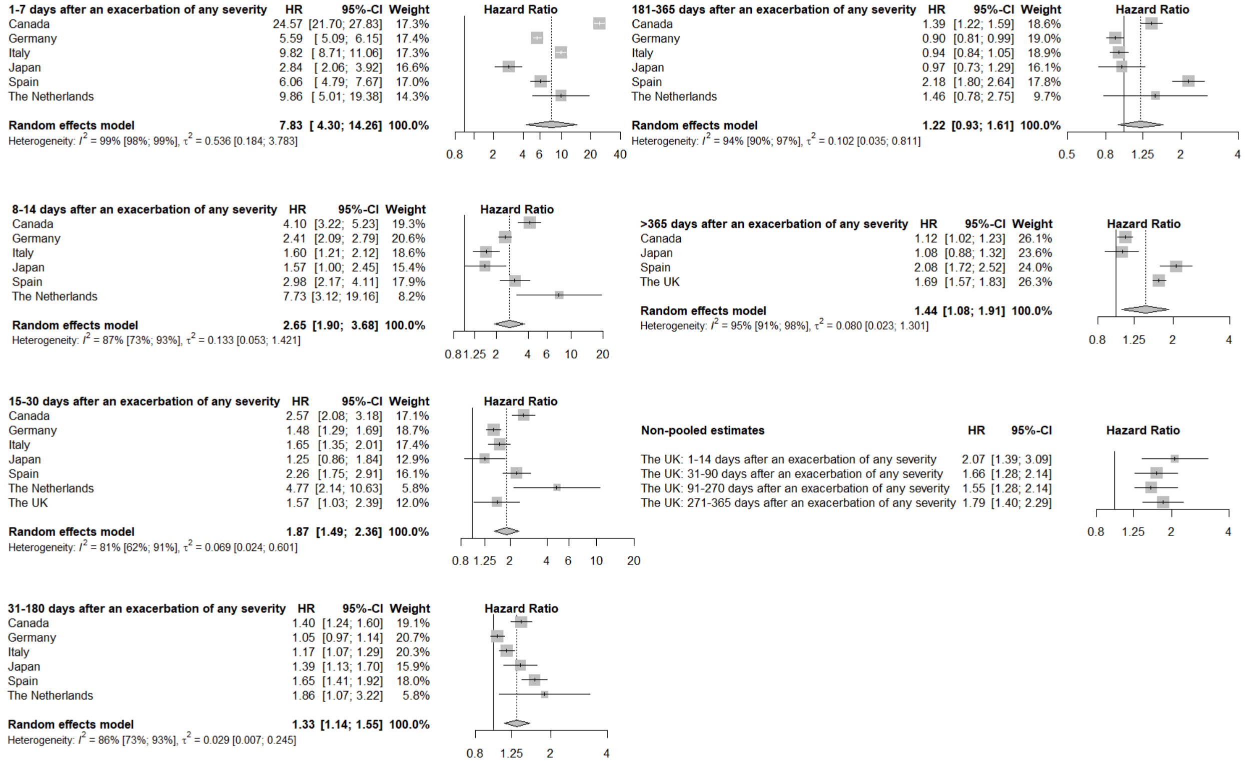

Supplementary Figure S7: Acute coronary syndrome after an exacerbation of any severity; statistical heterogeneity among study results

Higgins' and Thompson's I^2 statistic was computed to quantify the proportion of variation among estimated HRs that is due to between-study heterogeneity. The DerSimonian-Laird estimator [15] was used to quantify between-study variance τ^2, and the method of Jackson was used to compute the 95% CI for τ^2

Abbreviations: CI: confidence interval; CV: cardiovascular; HR: hazard ratio; UK: United Kingdom.

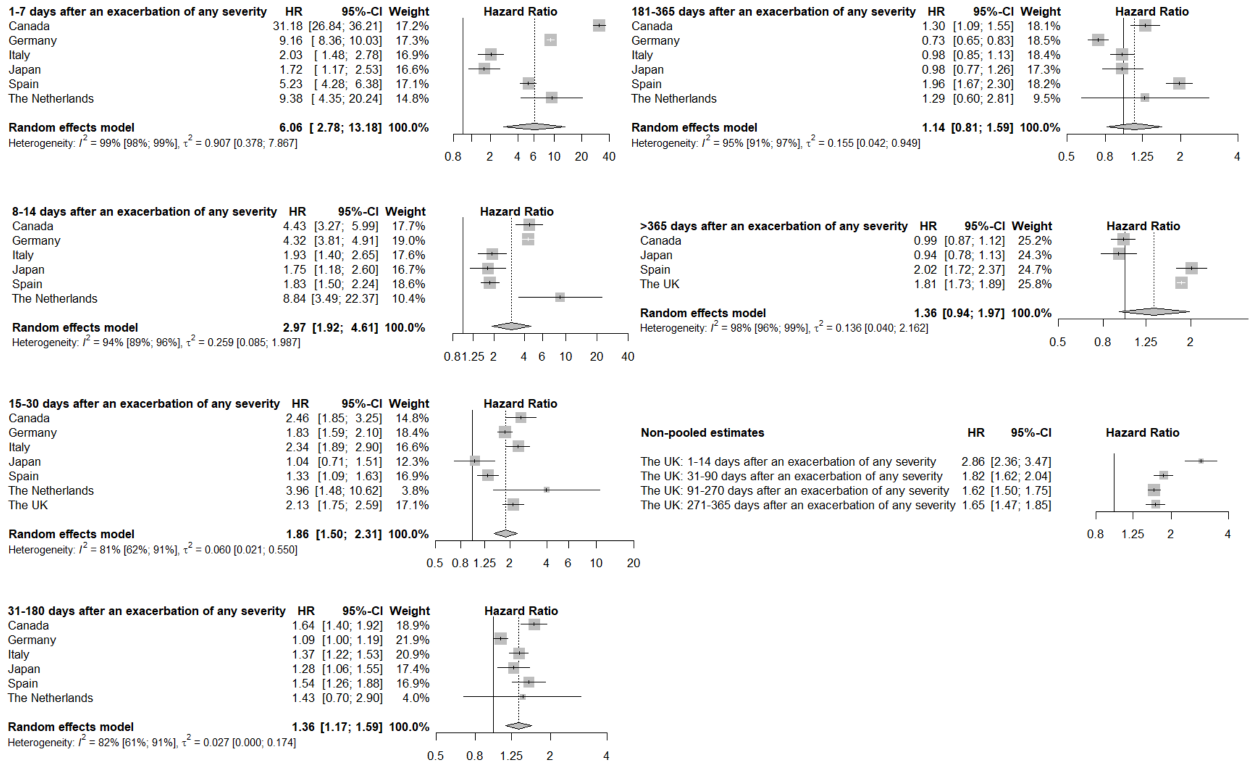

Supplementary Figure S8: Arrhythmia after an exacerbation of any severity; statistical heterogeneity among study results

Higgins' and Thompson's I^2 statistic was computed to quantify the proportion of variation among estimated HRs that is due to between-study heterogeneity. The DerSimonian-Laird estimator [15] was used to quantify between-study variance τ^2, and the method of Jackson was used to compute the 95% CI for τ^2

Abbreviations: CI: confidence interval; CV: cardiovascular; HR: hazard ratio; UK: United Kingdom.

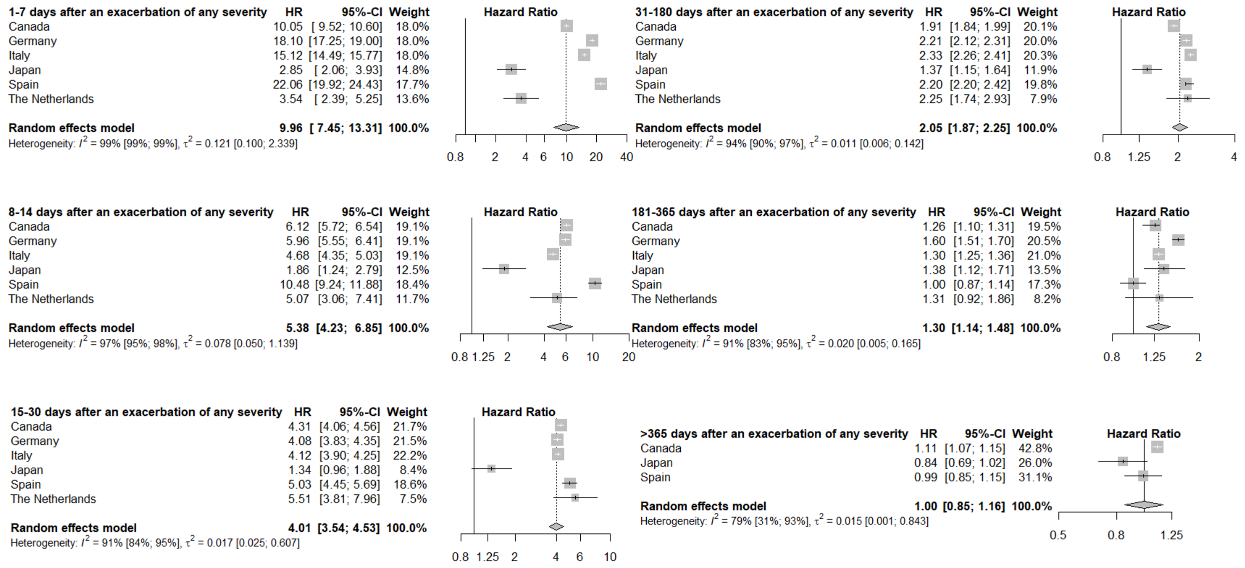

Supplementary Figure S9: All-cause death after an exacerbation of any severity; statistical heterogeneity among study results

Higgins' and Thompson's I^2 statistic was computed to quantify the proportion of variation among estimated HRs that is due to between-study heterogeneity. The DerSimonian-Laird estimator [15] was used to quantify between-study variance τ^2, and the method of Jackson was used to compute the 95% CI for τ^2

Abbreviations: CI: confidence interval; CV: cardiovascular; HR: hazard ratio.

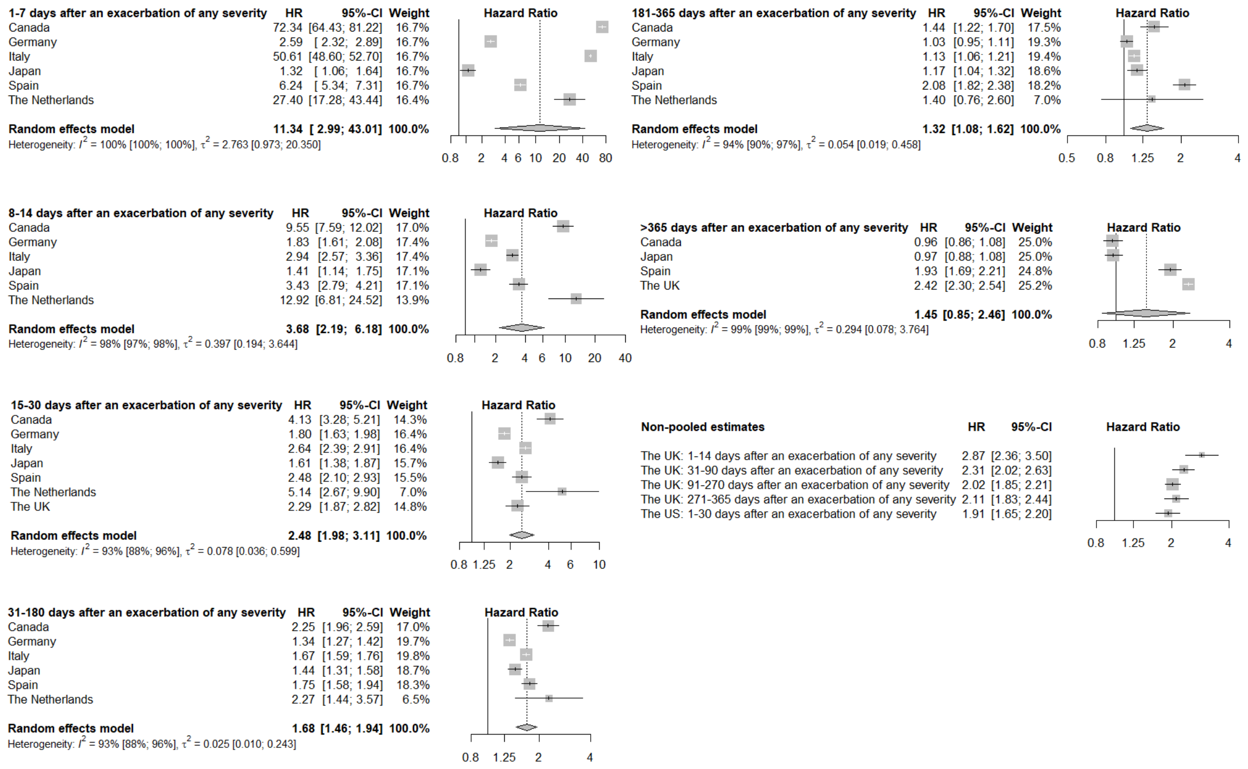

Supplementary Figure S10: Heart failure (decompensated) after an exacerbation of any severity; statistical heterogeneity among study results

Higgins' and Thompson's I^2 statistic was computed to quantify the proportion of variation among estimated HRs that is due to between-study heterogeneity. The DerSimonian-Laird estimator [15] was used to quantify between-study variance τ^2, and the method of Jackson was used to compute the 95% CI for τ^2

Abbreviations: CI: confidence interval; CV: cardiovascular; HR: hazard ratio; UK: United Kingdom; US: United States.

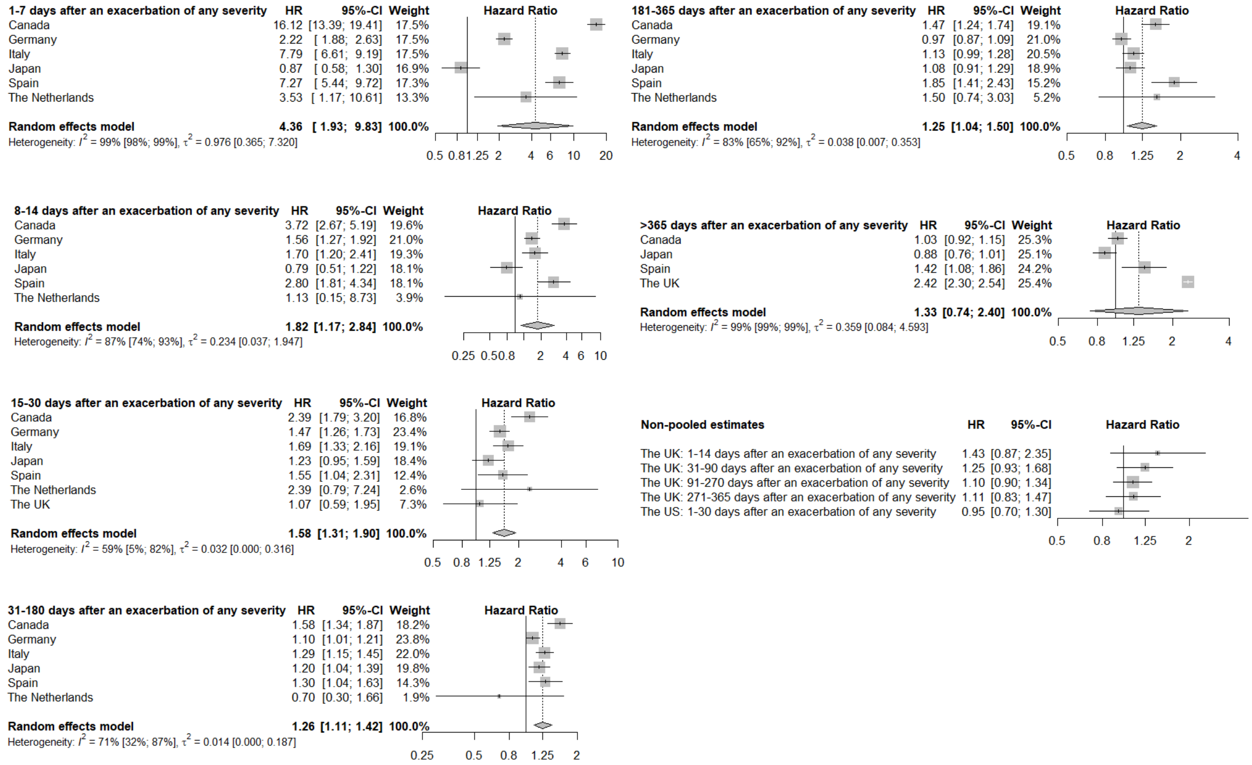

Supplementary Figure S11: Ischaemic stroke after an exacerbation of any severity; statistical heterogeneity among study results

Higgins' and Thompson's I^2 statistic was computed to quantify the proportion of variation among estimated HRs that is due to between-study heterogeneity. The DerSimonian-Laird estimator [15] was used to quantify between-study variance τ^2, and the method of Jackson was used to compute the 95% CI for τ^2

Abbreviations: CI: confidence interval; CV: cardiovascular; HR: hazard ratio; UK: United Kingdom; US: United States.

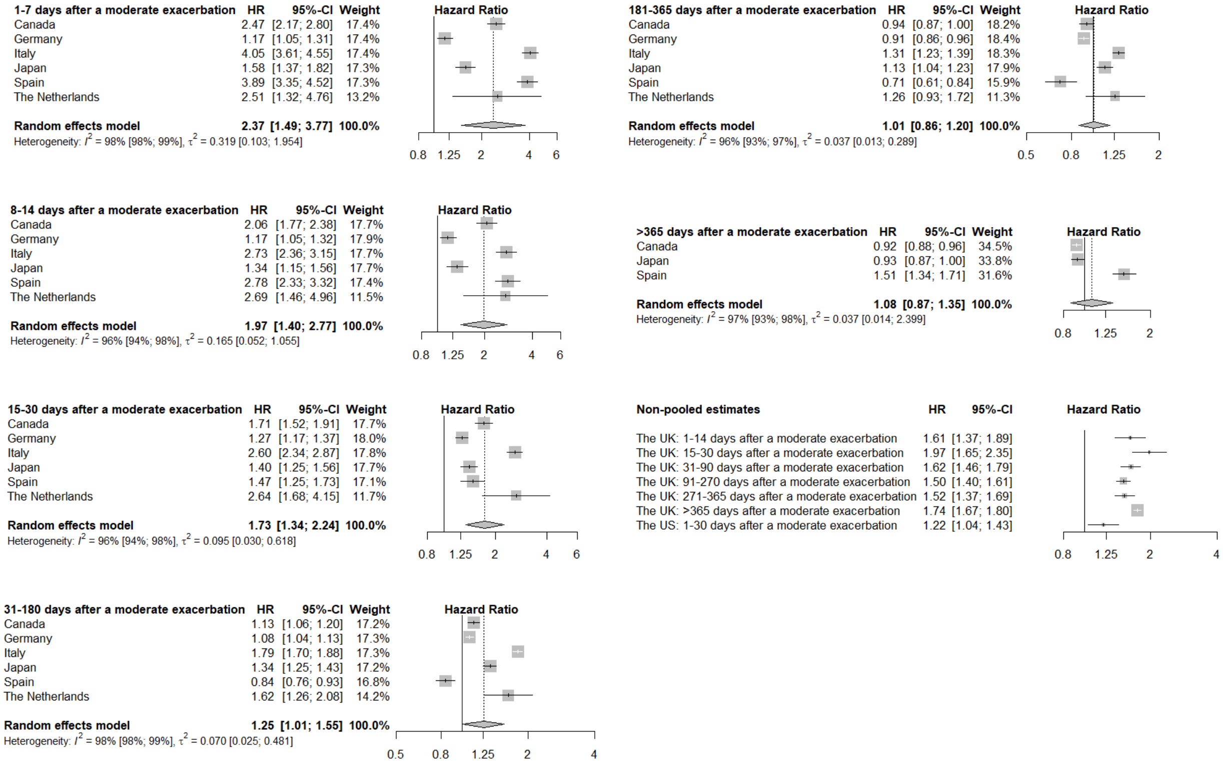

Supplementary Figure S12: Severe cardiovascular event including death after a moderate exacerbation; statistical heterogeneity among study results

Higgins’ and Thompson’s I^2 statistic was computed to quantify the proportion of variation among estimated HRs that is due to between-study heterogeneity. The DerSimonian-Laird estimator [15] was used to quantify between-study variance τ^2, and the method of Jackson was used to compute the 95% CI for τ^2

Abbreviations: CI, confidence interval, CV, cardiovascular, HR, hazard ratio, UK, United Kingdom, US, United States.

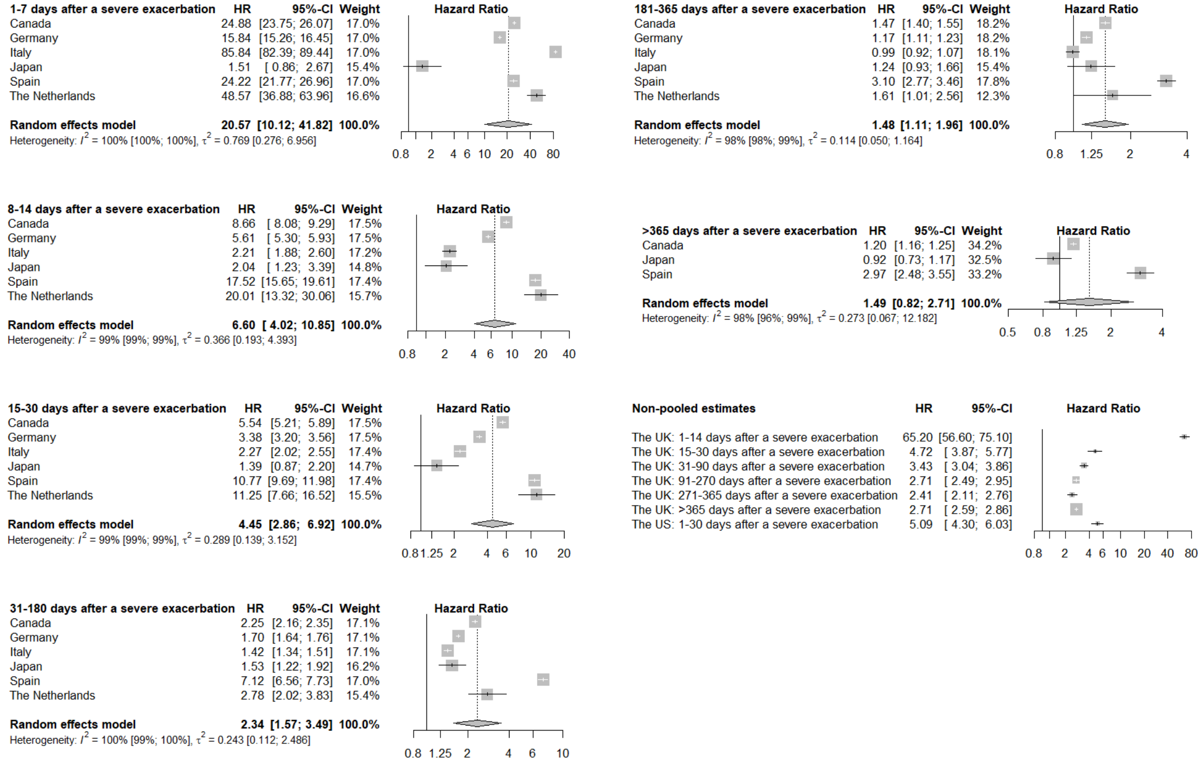

Supplementary Figure S13: Severe cardiovascular event including death after a severe exacerbation; statistical heterogeneity among study results

Higgins' and Thompson's I^2 statistic was computed to quantify the proportion of variation among estimated HRs that is due to between-study heterogeneity. The DerSimonian-Laird estimator [15] was used to quantify between-study variance τ^2, and the method of Jackson was used to compute the 95% CI for τ^2

Abbreviations: CI, confidence interval, CV, cardiovascular, HR, hazard ratio, UK, United Kingdom, US, United States.

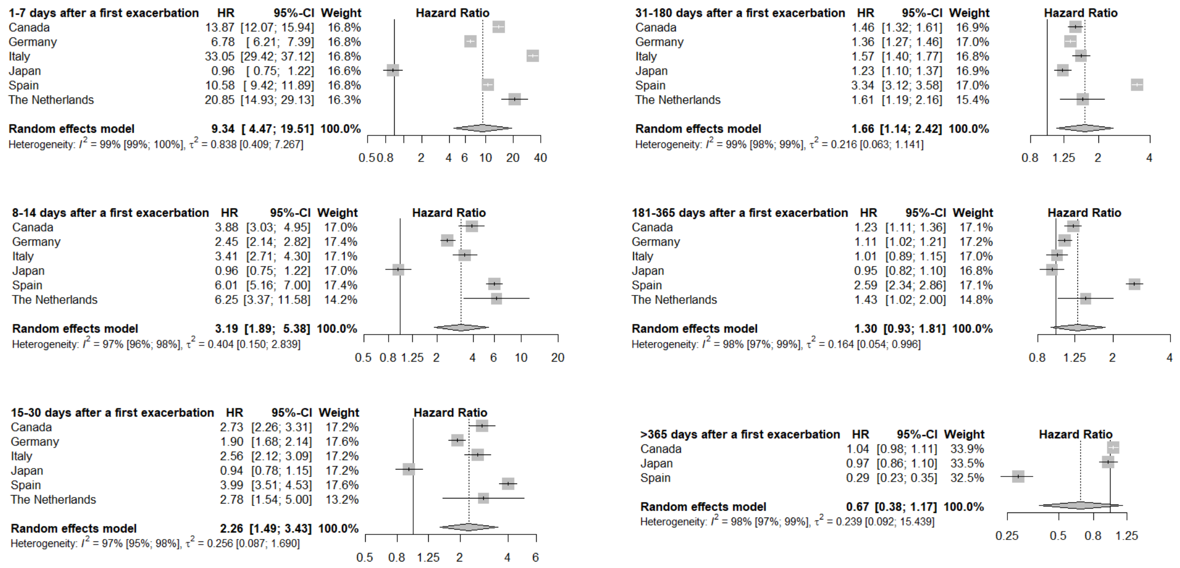

Supplementary Figure S14: Severe cardiovascular event including death after a first exacerbation; statistical heterogeneity among study results

Higgins' and Thompson's I^2 statistic was computed to quantify the proportion of variation among estimated HRs that is due to between-study heterogeneity. The DerSimonian-Laird estimator [15] was used to quantify between-study variance τ^2, and the method of Jackson was used to compute the 95% CI for τ^2

Abbreviations: CI, confidence interval, CV, cardiovascular, HR, hazard ratio.

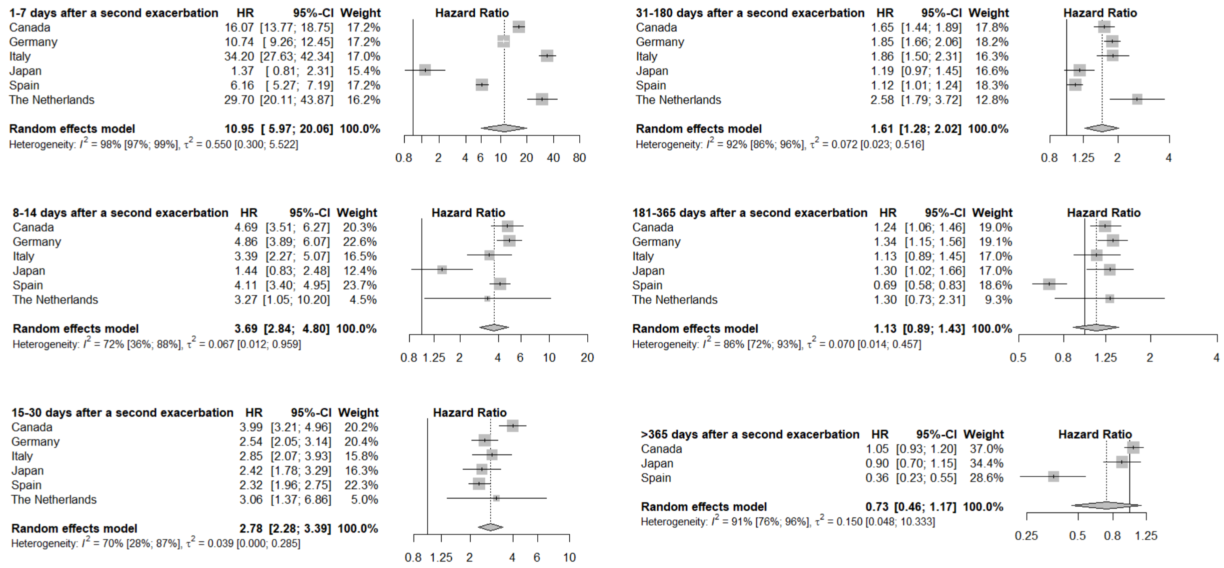

Supplementary Figure S15: Severe cardiovascular event including death after a second exacerbation; statistical heterogeneity among study results

Higgins' and Thompson's I^2 statistic was computed to quantify the proportion of variation among estimated HRs that is due to between-study heterogeneity. The DerSimonian-Laird estimator [15] was used to quantify between-study variance τ^2, and the method of Jackson was used to compute the 95% CI for τ^2

Abbreviations: CI, confidence interval, CV, cardiovascular, HR, hazard ratio.
